# Endogenous Trigeminal Neurons Induce Schwann Differentiation of Donor Stem Cells in Dental Pulp Regeneration via Reciprocal Extracellular Vesicles Interplay

**DOI:** 10.1002/exp2.70229

**Published:** 2026-10-08

**Authors:** Wanmin Zhao, Wenzhe Wang, Jia Guo, Jiani Liu, Wei Wang, Dihao Tao, Jiayi Dong, Zhonghan Li, Shiyu Liu, Songtao Shi, Tao Chen, Yan Jin, Bei Li

**Affiliations:** ^1^ State Key Laboratory of Oral & Maxillofacial Reconstruction and Regeneration National Clinical Research Center for Oral Diseases Shaanxi International Joint Research Center For Oral Diseases Center For Tissue Engineering School of Stomatology The Fourth Military Medical University Xi'an China; ^2^ Department of Stomatology Air Force Medical Center The Fourth Military Medical University Beijing China; ^3^ State Key Laboratory of Oral & Maxillofacial Reconstruction and Regeneration National Clinical Research Center for Oral Diseases Shaanxi Key Laboratory of Stomatology Department of Endodontics School of Stomatology The Fourth Military Medical University Xi'an China; ^4^ Center of Growth Metabolism and Aging Key Laboratory of Bio‐Resource and Eco‐Environment of Ministry of Education College of Life Sciences Sichuan University Chengdu China; ^5^ South China Center of Craniofacial Stem Cell Research Guanghua School of Stomatology Sun Yat‐sen University Guangzhou China; ^6^ Department of Anatomy and K.K. Leung Brain Research Centre The Fourth Military Medical University Xi'an China

**Keywords:** extracellular vesicles, mitochondria, schwann cells, tissue regeneration, trigeminal ganglion

## Abstract

The potential of stem cells transplantation to restore organ function in recipients is hindered by the poor integration of donor cells into host tissues. Here, we successfully regenerated tooth pulp in the root canal of minipigs by transplanting human deciduous tooth stem cells (SHED). The implanted SHED differentiated into several functional cells, particularly Schwann cells (SCs), which attracted and enwrapped regrown host axons in regenerated pulp. The reciprocal extracellular vesicles (EVs) communication between SHED and the host trigeminal ganglion (TG) regulated the regeneration of dental nerves. EVs derived from SHED (SHED‐EVs) facilitated axonal growth, while those released by TG neurons (TGN) induced the differentiation of SHED into SCs. TGN that engulfed SHED‐EVs secreted endogenous EVs (ST‐EVs) and migrated to the regenerated pulp tissue. ST‐EVs from TGN are enriched with TUFM (Tu translation elongation factor, mitochondrial), which, in turn promotes Schwann cell differentiation (SC differentiation) of SHED. This study highlights the importance of cellular signal exchange during the integration of donor cells and host tissues for organ regeneration.

## Graphical abstract

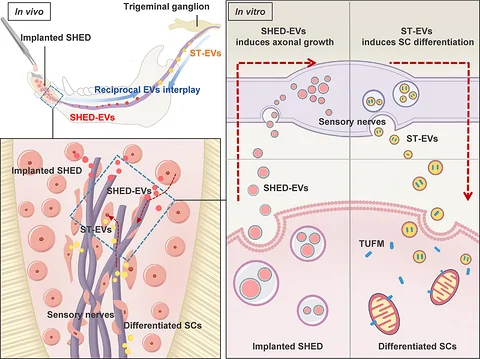

## 1 Introduction

Allogeneic donor cells transplantation for tissue regeneration holds great promise as a therapeutic strategy for repairing various organs. Clinical trials involving the transplantation of donor‐derived cells have successfully restored the structure and function of host tissues in humans [1, 2, 3, 4, 5]. Implanted stem cells are believed to regenerate tissues by self‐renew and multi‐differentiation, which corporate with recruited host derived neurovascular bundles to achieve functional regeneration [6, 7]. Nevertheless, the poor integration of donor cells into host tissues poses numerous challenges that impedes the development of novel strategies aimed at enhancing functional restoration [8, 9, 10]. Therefore, it is crucial to gain a comprehensive understanding of the similarities and differences between donor‐derived cells and endogenous host cells, as well as the fate behavior of transplanted cells [11, 12].

Dental pulp regeneration offers a useful model for investigating donor–host interactions, as implanted stem cells are housed in a protective chamber that shields them from host‐derived stimuli [13]. In human dental pulp, peripheral‐related SCs not only engage in dental innervation, but also form a multicellular glial network at the dentin‐pulp interface that is crucial for pathogen resistance and dentin repair [14]. During dental development, a significant population of mesenchymal stem cells (MSCs) originate from nerve‐related SCs, which further give rise to various dental pulp cells after being innervated by trigeminal nerve fibers [15, 16]. Recently, as the spatiotemporal relationship and regulatory mechanisms during dental development are further elucidated, it has been revealed that dental pulp regeneration recapitulates its developmental trajectory [17, 18]. As MSCs co‐cultured with neurons differentiate into SCs [19], we speculate whether the implanted MSCs will acquire an SC‐like phenotype and cooperate with host‐derived dental nerves to regenerate dental pulp.

Sensory nerves derived from TG are proved to regulate tooth development and homeostasis [20, 21, 22]. In previous studies, we had achieved pulp regeneration in humans using autologous SHED, leading to restoration of tooth sensation and promotion of tooth development [23]. Therefore, we speculated that functional dental pulp regeneration requires implanted cells to be innervated and regulated by sensory nerves, a process mediated by reciprocal communication between the implanted cells and host TG. Extracellular vesicles (EVs) play a significant role in mediating intercellular communication, which can traverse the interstitial space and ultimately reach the circulation, exerting local paracrine or distal systemic effects [24, 25]. Although MSCs derived EVs are well established in promoting axon regeneration [26], a recent finding is that EVs are engulfed by neurons at nerve endings and transported retrogradely to the neuron body [27]. After processing in endosomes, the EVs are further delivered to next‐level neurons, which connect the long‐distance neural network [28, 29]. Hence, we speculate that the donor cells promoting endogenous axon regeneration and host TG regulating the fate behavior of donor cells during dental pulp regeneration are mediated by reciprocal EV communication.

Here we successfully achieved the heterologous regeneration of dental pulp in minipigs and rats through SHED implantation. Single‐cell RNA sequencing (scRNA‐seq) analysis demonstrated that a significant group of implanted SHED differentiated into SCs, which seamlessly surrounded and integrated with the host axons. We observed that EVs derived from SHED (SHED‐EVs) promoted axonal growth by migrating to the TGN. Moreover, after internalizing SHED‐EVs, TGN could secrete EVs (ST‐EVs) retrogradely to the regenerated pulp tissue. Inhibition of endogenous ST‐EVs secreted from TG by GW4869 impaired SC differentiation in regenerated dental nerves. Mechanistically, SHED‐EVs promoted axonal growth of TGN, and ST‐EVs from TGN promoted SC differentiation of SHED subsequently by delivering TUFM (Tu translation elongation factor, mitochondrial). These findings highlight the intricate intercellular communication through EVs during the integration of donor and host cells in functional tissue regeneration.

## 2 Results

### 2.1 SHED Implantation Regenerates Dental Pulp With SCs Enwrapped Nerves

In our previous study, we successfully utilized autologous human tooth stem cells to regenerate the entire pulp in humans, leading to the recovery of tooth sensation and promotion of tooth development [23]. To understand the mechanisms for the implanted donor tooth stem cells to regenerate dental pulp, SHED were isolated as previously described [30], and implanted into empty minipig incisor root canals with pulpectomy for chimeric human‐pig pulp regeneration (Figure 1A). Three‐dimensional (3D) reconstruction of the normal teeth and SHED implanted teeth of minipigs after 6 months were scanned by micro‐computed tomography (micro‐CT). Representative 3D images of SHED implanted teeth showed the similar root canal morphology with normal minipig teeth. Hematoxylin and Eosin (HE) staining exhibited whole pulp tissue regeneration with odontoblast layer and blood vessels after SHED implantation (Figure 1B,C). Both normal and regenerated minipig pulps were innervated by full‐length βIII tubulin (βIII Tub)‐immunopositive axons, which were enwrapped by S100b‐ and MBP‐immunopositive SCs (Figure 1D,E and Figure S1A). The axons in normal and regenerated pulp expressed sensory nerve markers such as CGRP, TRPV1, and TRPM8 (Figure S1B). These data prove that heterologous SHED implantation successfully regenerates dental pulp in minipigs.

**FIGURE 1:**
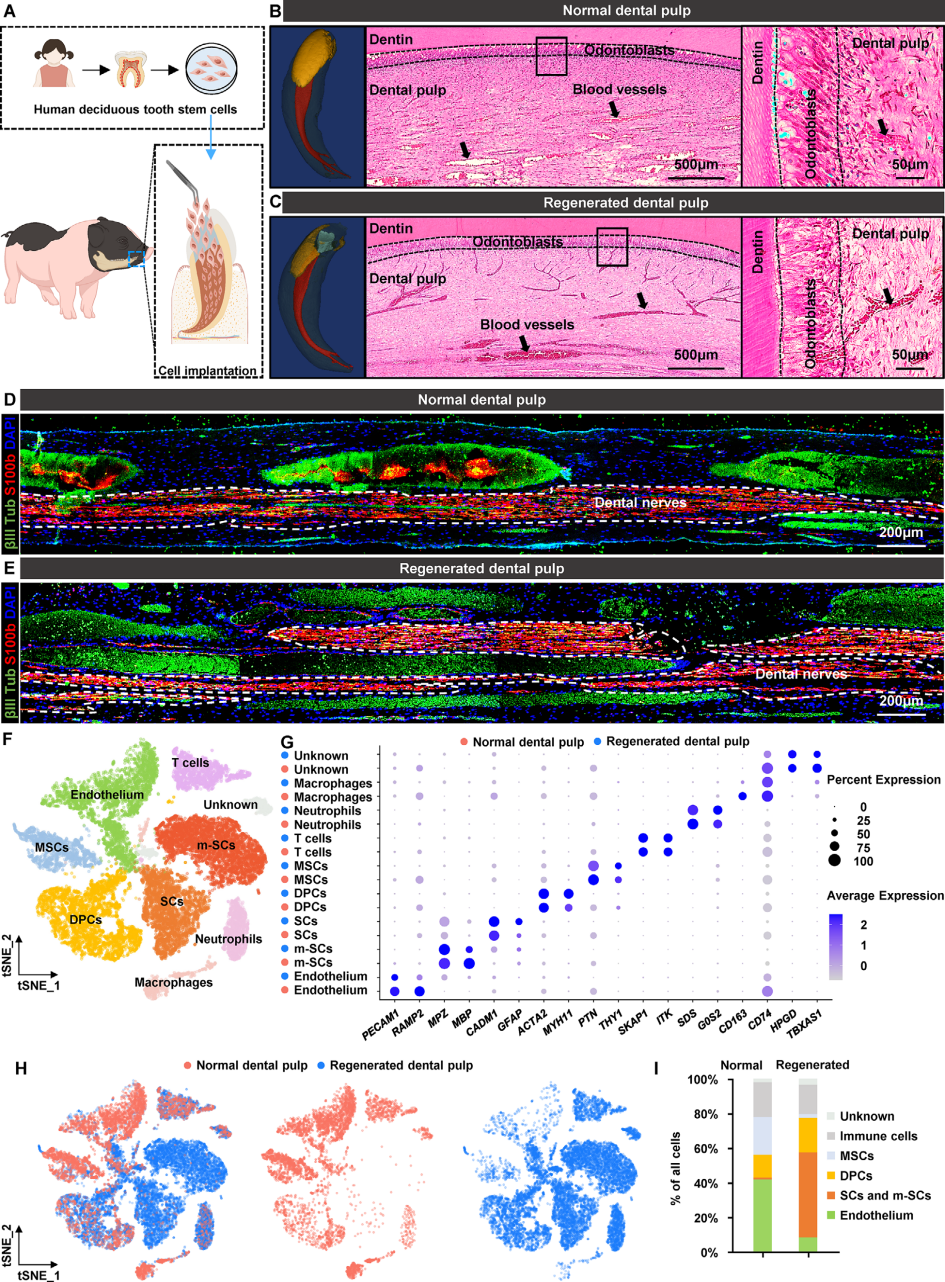
SHED implantation regenerates dental pulp with SCs enwrapped nerves. (A) Schematic drawings of dental pulp regeneration in minipigs with SHED implantation. (B and C) Micro‐CT scanning (left) and HE staining (center and right) for normal (B) and regenerated dental pulp after SHED implantation for 6 months (C). Odontoblast layer (black dotted lines) and blood vessels (black arrowheads) is observed. The black box at the margin of dental pulp is magnified in right panel. Scale bar, 500 µm in the center panel and 50 µm in the right panel. (D and E) Immunostaining of axons and SCs in normal (D) and regenerated minipig dental pulp (E). White dotted lines indicate SCs (S100b) enwrapped axons (βIII Tubulin, βIII Tub). Scale bar, 200 µm. (F) Dimensionality reduction visualization by t‐SNE plot of cell clusters in normal and regenerated minipig dental pulps at 6 months after SHED implantation using scRNA‐seq. The cells were isolated from three normal and regenerated dental pulp tissues respectively for scRNA‐seq. (G) Dotplot of featured genes of cell clusters in normal and regenerated minipig dental pulps at 6 months after SHED implantation. (H) t‐SNE visualization of single‐cell transcriptomic profiles of normal and regenerated minipig dental pulps. Cells are distinguished by different colors. Separated view of normal and regenerated cell clusters are shown. (I) Cellular composition of normal and regenerated minipig dental pulps based on clusters identified in (F).

To investigate the heterogeneity of cells in both normal and regenerated dental pulp in vivo, we performed scRNA‐seq on normal pig dental pulp tissues and regenerated dental pulp tissues at six months following SHED implantation. Dimensionality reduction of the transcriptomes was visualized using t‐distributed stochastic neighbor embedding (t‐SNE) analysis. Louvain clustering identified nine prominent cell clusters in both normal and regenerated dental pulp, which were assigned to endothelium (*PECAM* ^+^/*RAMP2* ^+^), myelinated Schwann cells (m‐SCs, *MPZ^+^*/*MBP^+^*) and SCs (*CADM1* ^+^/*GFAP* ^+^), dental pulp cells (DPCs, *ACTA2* ^+^/*MYH11* ^+^), MSCs (*PTN* ^+^/*THY1* ^+^), T cells (*SKAP1* ^+^/*ITK* ^+^), neutrophils (*SDS* ^+^/*G0S2* ^+^), macrophages (*CD163* ^+^/*CD74* ^+^), and unknown cells (*HPGD* ^+^/*TBXAS1* ^+^) by their distinct marker expressions (Figure 1F,G). Using dotplot and heatmap, we identified various top‐enriched genes and unique markers specific to each cell cluster. We observed that the same signature genes were expressed in both normal and regenerated dental pulps (Figure 1G and Figure S1C). However, the proportions of the different cell types within the two tissues were not identical (Figure 1H,I). In regenerated dental pulp, approximately 49.3% of the cells expressed SC‐related markers, in contrast to only 1.0% of SCs in normal dental pulp (Figure 1H,I). These findings demonstrate that the cell populations in the regenerated dental pulp, following SHED implantation, closely resemble those observed in normal dental pulp. Notably, SCs, which are predominant in the regenerated tissue, likely play a crucial role in the re‐establishment of innervation and restoring the sensory function of the dental pulp.

### 2.2 Implanted SHED Integrate With Host Endogenous Cells in a Feedback‐Driven Manner to Regenerate Dental Pulp

To observe the regenerative progression of dental pulp in detail, we examined the regenerated dental pulp at 3 and 6 months after SHED implantation. HE staining indicated that both the odontoblast layer and blood vessels were regenerated in dental pulp (Figure 2A). Furthermore, comparisons of the regenerated dental pulp nerves at 3 and 6 months after SHED implantation showed an increase in the intensity of regenerated axons as well as a greater distribution of SCs over time (Figure 2B,C). We conducted scRNA‐seq analysis on the regenerated dental pulp at both time points to comprehensively characterize their cellular and molecular landscape. Remarkably, both samples were composed of nine distinct cell populations (Figure 2D). Since the fate of implanted stem cells during dental pulp regeneration has not been fully deciphered, we distinguished the implanted human cells from endogenous host cells to identify their progeny. t‐SNE analysis revealed that the cellular components of the regenerated dental pulp at 3 and 6 months after SHED implantation included implanted and endogenous cells (Figure 2E). The implanted cells at both 3 and 6 months after SHED implantation gave rise to DPCs, endothelium, MSCs, and SCs, all of which expressed similar signature genes according to their cell types (Figure 2F,G and Figure S2A). We also validate whether these cells were derived from implanted SHED by implanting GFP^+^ SHED in minipigs for dental pulp regeneration. Immunofluorescence staining showed that the GFP labeled SHEDs were differentiated into SCs, odontoblasts, and endothelial cells, respectively (Figure S2B). In the 3 month implantation group, 19.9% of the implanted cells were identified as MSCs, while 3.7% were SCs and m‐SCs. The recruited host cells were predominantly MSCs (18.7%) and endothelium (15.3%) (Figure 2H). However, in the 6 month implantation group, only 0.4% of the implanted cells were identified as MSCs, whereas 19.0% expressed SC‐related markers. Host‐derived cells primarily consisted of SCs and m‐SCs (29.7%) and DPCs (15.5%) at 6 months implantation (Figure 2H). These findings suggest that dental pulp regeneration relies on the integration of implanted and endogenous cells. A significant population of SCs and m‐SCs in the regenerated dental pulp is likely derived from the implanted SHED.

**FIGURE 2:**
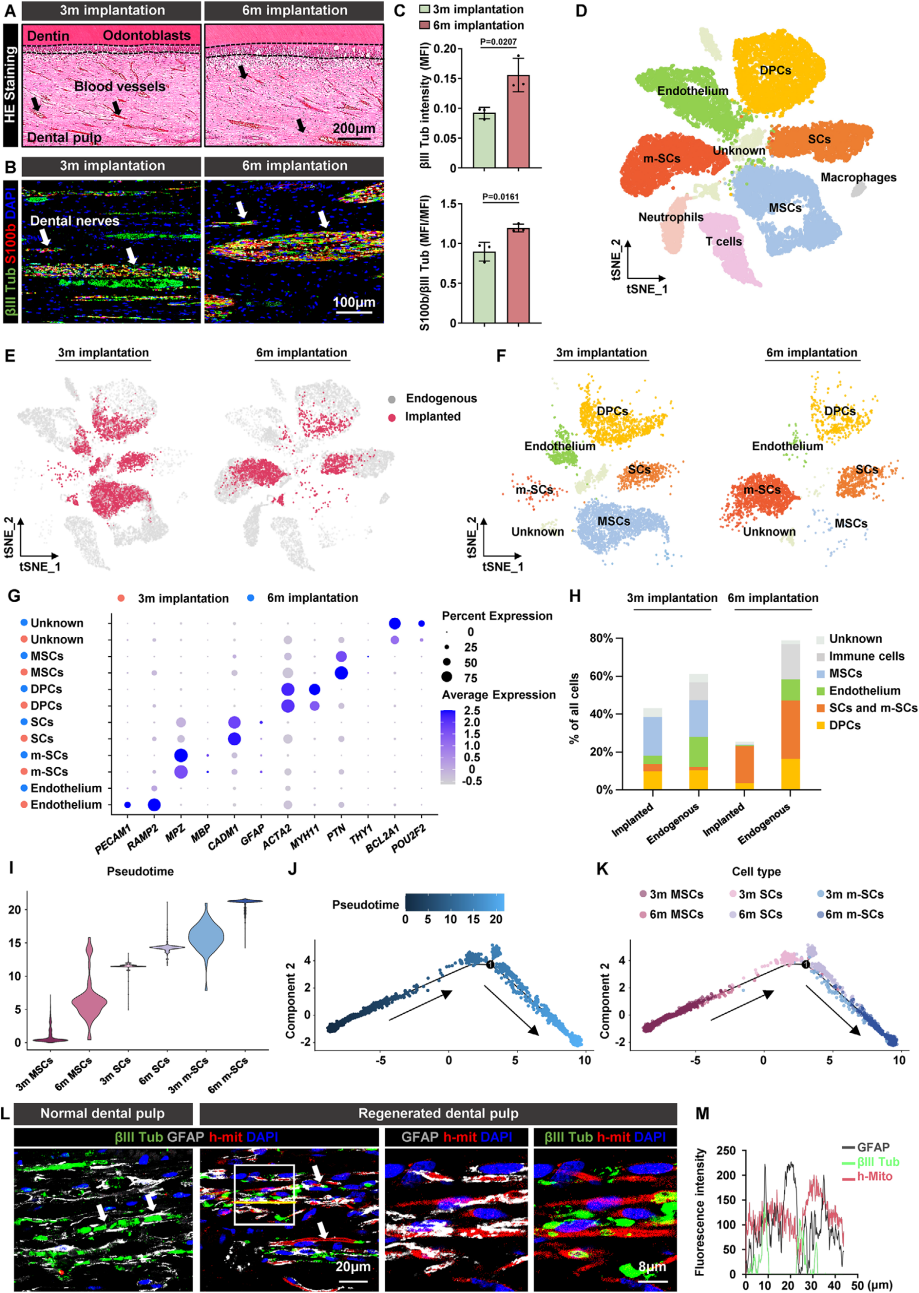
Implanted SHED integrate with host derived endogenous cells to regenerate dental pulp. (A) HE staining for regenerated dental pulp at 3 and 6 months after SHED implantation. Odontoblast layer (black dotted lines) and blood vessels (black arrowheads) is observed. Scale bar, 200 µm. (B) Immunostaining for axons and SCs of regenerated dental pulp at 3 and 6 months after SHED implantation. White arrowheads indicate SCs (S100b) enwrapped regenerated axons (βIII Tub). Scale bar, 100 µm. (C) Quantification of regenerated axons and SC differentiation in (B). Error bars denote SD from three minipig dental pulps per condition. Statistics by unpaired *t* test. (D) Dimensionality reduction visualization by t‐SNE plot of cell clusters in regenerated dental pulp at 3 and 6 months after SHED implantation using scRNA‐seq. The cells were isolated from three regenerated dental pulp tissues in minipig incisors at 3 and 6 months after SHED implantation respectively for scRNA‐seq. (E) t‐SNE visualization of single‐cell transcriptomic profiles of cell origination of regenerated dental pulp at 3 and 6 months after SHED implantation. Cell originations are labeled by different colors. (F) Louvain clustering and annotation of cellular components from implanted cells in regenerated dental pulp at 3 and 6 months after SHED implantation. (G) Dotplot of featured genes of cell clusters derived from implanted cells in regenerated dental pulp at 3 and 6 months after SHED implantation. (H) Cellular composition of regenerated dental pulp at 3 and 6 months after SHED implantation based on clusters identified in (F). Cells are classified by implanted and endogenous derivation. (I) Volin plots of pseudotime status of MSCs, SCs and m‐SCs derived from implanted cells in regenerated dental pulp at 3 and 6 months after SHED implantation. (J and K) Pseudotime analysis by Monocle2 revealed the developmental trajectories of MSCs, SCs and m‐SCs. Pseudotime increases in the direction indicated by the black arrowheads. (L and M) Normal and regenerated dental pulps from minipig were stained for βIII Tub, GFAP, h‐mit and DAPI (L). Fluorescence intensity profiles (M) for GFAP (white), βIII Tub (green) and h‐mit (red) were obtained along the indicated solid yellow line. White arrowheads indicate SCs in both normal and regenerated minipig dental pulps. The white box in regenerated dental pulp image is magnified in the right panel. Scale bar, 20 µm in the left two panel and 8 µm in the right two panel.

To better understand the potential origin and differentiation processes of SCs derived from implanted cells in regenerated dental pulp, pseudotime trajectory analysis was conducted using Monocle2. MSCs, SCs, and m‐SCs derived from implanted cells were predicted to be at an earlier stage in the 3‐month implantation group compared to the 6‐month group (Figure 2I). The trajectories revealed that MSCs were primarily concentrated at the beginning, m‐SCs at the end, and SCs at an intermediate position (Figure 2J,K). The signature genes of these cell types were also analyzed throughout the pseudotime trajectory. As expected, the expression of *PTN* and *PTPRD* in MSCs decreased along the trajectory, while markers for SCs (*CADM1* ^+^/*NAV2* ^+^) and m‐SCs (*MPZ* ^+^/*PMP22* ^+^) increased sequentially (Figure S3A). These data indicate that SCs differ from implanted SHED.

In normal dental pulp, SCs tightly envelop sensory nerves extending from the TG [31]; however, the source of sensory nerves in regenerated dental pulp remains unidentified. Since mitochondria are predominantly found in axons [32], human mitochondria (h‐mit) were used to label SHED derived cells. The results indicated that h‐mit were co‐expressed with GFAP labeled SCs but not with the axons, which verified that the axons were derived from the host TG (Figure 2L,M). Moreover, CellChat analysis was performed to explore the complex interactions between implanted and host cells during dental pulp regeneration. In the 3 month implantation group, MSCs derived from implanted cells exhibited strong interactions with various endogenous cells (Figure S3B). They not only sent autocrine signals to themselves but also provided critical signals to other implanted and recruited endogenous cells (Figure S3C). In the 6 month implantation group, the interaction activity among implanted cells decreased; however, the implantation‐derived MSCs coordinated with various endogenous cells to dominate outgoing signals (Figure S3D,E). These data indicate that dental nerve regeneration depends on the integration of host TG with differentiated SCs from implanted SHED, and the communication between implanted SHED and host endogenous cells is feedback‐driven.

### 2.3 SHED‐EVs Trigger Host Axonal Growth and Dental Nerve Regeneration

As the regenerated dental nerves were composed with implanted SHED derived SCs and host TG derived sensory nerves, we hypothesized that these regrew host derived axons in dental pulp may be guided by implanted SHED. EVs has been reported as a new aspect of transcellular signaling, which could deliver various bioactive molecules like proteins, nucleic acid and lipid [33]. Our previous study demonstrated that SHED‐EVs positively impacted dental pulp regeneration [34, 35]. However, the specific influence of SHED‐EVs on dental nerve regeneration remains unknown. In the current study, SHED were pretreated with GW4869, an inhibitor for EVs secretion, prior to implantation (GW4869‐SHED implantation) to confirm the importance of SHED‐EVs in dental nerve regeneration. After GW4869‐SHED implantation, the intensity of the regenerated axons was reduced, and the differentiation of SCs was impaired compared to control group after DMSO‐SHED implantation (Figure 3A,B and Figure S4A,B).

**FIGURE 3:**
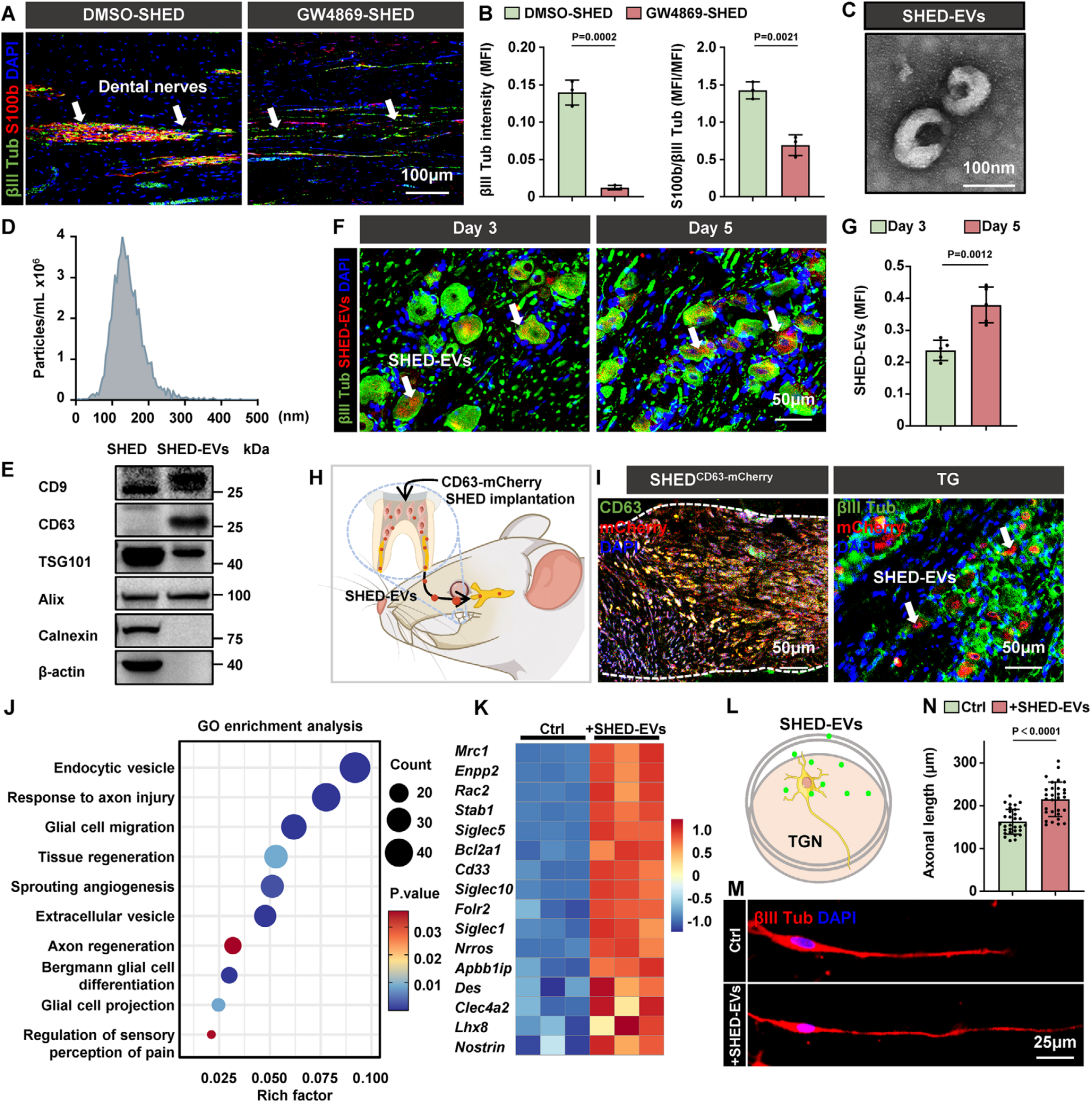
SHED‐EVs trigger host axonal growth and dental nerve regeneration. (A and B) Immunostaining (A) and quantification (B) for regenerated axons and SCs at 6 months after DMSO‐SHED or GW4869‐SHED implantation. White arrowheads indicate SCs enwrapped regenerated axons. Scale bar, 100 µm. Error bars denote SD from three regenerated minipig dental pulps per condition. Statistics by unpaired *t* test. (C to E) The characterization of SHED‐EVs, TEM analysis of SHED‐EVs (C), NTA analysis of SHED‐EVs (D), western blot analysis for the expression of SHED‐EVs positive markers CD9, CD63, TSG101 and Alix and positive marker Calnexin (E). Scale bar, 100 nm. (F and G) Immunostaining (F) and quantification (G) of in vivo tracing of PKH26‐labeled SHED‐EVs (red) at 3 and 5 days after injection. Scale bar, 50 µm. Error bars denote SD from five rats TGs. Statistics by unpaired *t* test. (H) Schematic drawings of rat dental pulp regeneration with CD63‐mCherry SHED implantation. (I) Immunostaining of in vivo tracing SHED‐EVs during dental pulp regeneration following the implantation of CD63‐mCherry SHED. Left panel shows the co‐localization of CD63 (green) and mCherry (red) in regenerated rat dental pulp. Right panel shows the mCherry (red) signals in TG. White arrowheads indicate mCherry positive puncta in TG. Scale bar, 50 µm. (J) GO analysis of significantly upregulated genes in TGN after internalizing SHED‐EVs. The Y‐axis represents GO terms and the X‐axis represents rich factor. (K) Upregulated genes involved in axon regeneration of TGN after internalizing SHED‐EVs were shown. *n* = 3/group. (L to N) The effect of SHED‐EVs on axonal growth of TGN. SHED‐EVs (30 µg ml^−1^) were added into DIV2 primary TGN for 24 h (L). Immunostaining (M) and quantification (N) of TGN axonal length. Scale bar, 25 µm. Error bars denote SD from thirty TGNs per condition. Statistics by unpaired *t* test.

A novel rat model for dental pulp regeneration was constructed by implanting SHED into the root canals of the maxillary first molar to determine the mechanism by which SHED‐EVs facilitate dental nerve regeneration. The implanted SHED not only achieved the regeneration of dental pulp tissues but also differentiated into SCs during rat dental pulp regeneration, which was consistent with the minipig's result (Figure S4C–E). SHED‐EVs were isolated and characterized for morphology, diameters, and surface markers. SHED‐EVs were presented as spherical vesicles with a bilayer membrane structure (average diameter, 143.5 nm) (Figure 3C,D) and expressed typical EVs markers (positive for CD9, CD63, TSG101, and Alix and negative for Calnexin) (Figure 3E). The transport of SHED‐EVs during dental nerve regeneration was observed via an in vivo imaging assay after the injection of DiR‐labeled SHED‐EVs into the root canals of the maxillary first molar of rats. SHED‐EVs were transported into the ipsilateral TG and accumulated in a time‐dependent manner (Figure S4F,G). The accumulation of SHED‐EVs in the soma of TGN was observed by injecting PKH26‐labeled SHED‐EVs, either (Figure 3F,G). Considering the dye aggregation and membrane transfer, SHED were transfected with Lentivirus‐CD63‐mCherry before implantation to specifically label intracellular endosomal intraluminal vesicles of secreted EVs during pulp regeneration in vivo (Figure 3H). As expected, CD63‐mCherry‐positive signals in regenerated dental pulp were also detected in the soma of TGN after 6 weeks of CD63‐mCherry SHED implantation (Figure 3I). Collectively, these findings confirm that SHED‐EVs migrate to TG and are engulfed by TGN during the dental pulp regeneration process.

Moreover, PKH26‐labeled SHED‐EVs were observed to be engulfed by primary cultured TGN in vitro (Figure S5A). To further know the function of TGN after internalization of SHED‐EVs, RNA sequencing (RNA‐seq) was performed to analyze the gene expression profiles of TGN with or without internalization of SHED‐EVs. Gene ontology (GO) enrichment analysis based on RNA‐seq showed a significant enrichment of axon regeneration‐associated genes in TGN with internalization of SHED‐EVs undergoing the most pronounced changes (Figure 3J). A total of 391 genes were upregulated and 36 genes were downregulated in TGN with internalization of SHED‐EVs (Figure S5B). SHED‐EVs treatment predominantly upregulated the expression of genes involved in axon regeneration (Figure 3K), indicating a possible promotion of axonal growth in TGN with internalization of SHED‐EVs. To evidence that, SHED‐EVs were directly added to primary cultured TGN, which significantly boosted axonal growth (Figure 3L–N). The function of SHED‐EVs was further verified by coculturing SHED with TGN in a transwell, while the TGN axonal growth was reduced when the secretion of SHED‐EVs was inhibited by GW4869 or shRAB27A transfection (Figure S5D–I). Our results demonstrate that SHED‐EVs trigger host axonal growth in dental nerve regeneration.

### 2.4 Reciprocal EVs Communication Between Donor SHED and Host TG in Dental Nerve Regeneration

We have verified that GW4869‐SHED implantation abolished axonal regeneration and hindered SC differentiation during minipig pulp regeneration. While SHED‐EVs have been shown to promote axonal regeneration, the underlying mechanisms by which inhibiting SHED‐EVs secretion affects SC differentiation remain unclear. To primarily address this question, TGN was directly co‐cultured with SHED for 2, 4, 6, and 8 weeks. The axonal density progressively increased and peaked at the sixth week. However, the differentiation of SHED into SCs consistently increased until the eighth week (Figure S6A,B). To further explore the temporal sequence during dental nerve regeneration in vivo, the co‐cultured cells were implanted into an empty tooth root scaffold and transplanted subcutaneously into immunocompromised mice. The intensity of regenerated axons plateaued in the sixth week, while SC differentiation continued to increase until the eighth week (Figure S6C,D). These findings suggest that the completion of axonal growth is earlier than that of SC differentiation in the dental cavity, thus implying a possible sequential relationship that the regenerated axons induce SC differentiation subsequently.

We have shown that SHED‐EVs were transported into TGN to boost axonal regeneration. GO enrichment analysis also showed notable effects on endocytic vesicles and extracellular vesicles in TGN with SHED‐EVs internalization (Figure 3J). Notably, the top 15 significant enrichments of GO cellular components indicated a possible EVs activity in TGN with SHED‐EVs internalization (Figure S5C). Moreover, gene‐set enrichment analysis (GSEA) confirmed a significant enrichment of the signaling events associated with multivesicular body, endocytic vesicle membrane, and regulation of vesicle mediated transport in TGN after internalizing SHED‐EVs (Figure 4A–C). Therefore, we hypothesized that TGN‐derived EVs (TGN‐EVs) retrograded back toward the implanted SHED and regulated the fate of these implanted cells. This hypothesis was verified by injecting AAV9‐hsyn‐CD63‐mCherry into TG to label the secretion of neural EVs in vivo (Figure 4D). With remarkable labeling of CD63‐mCherry in the TG, the mCherry positive signals were also observed in implanted SHED, confirming the successful delivery of TGN‐EVs to implanted SHED, which may participate in the fate regulation of SHED (Figure 4E).

**FIGURE 4:**
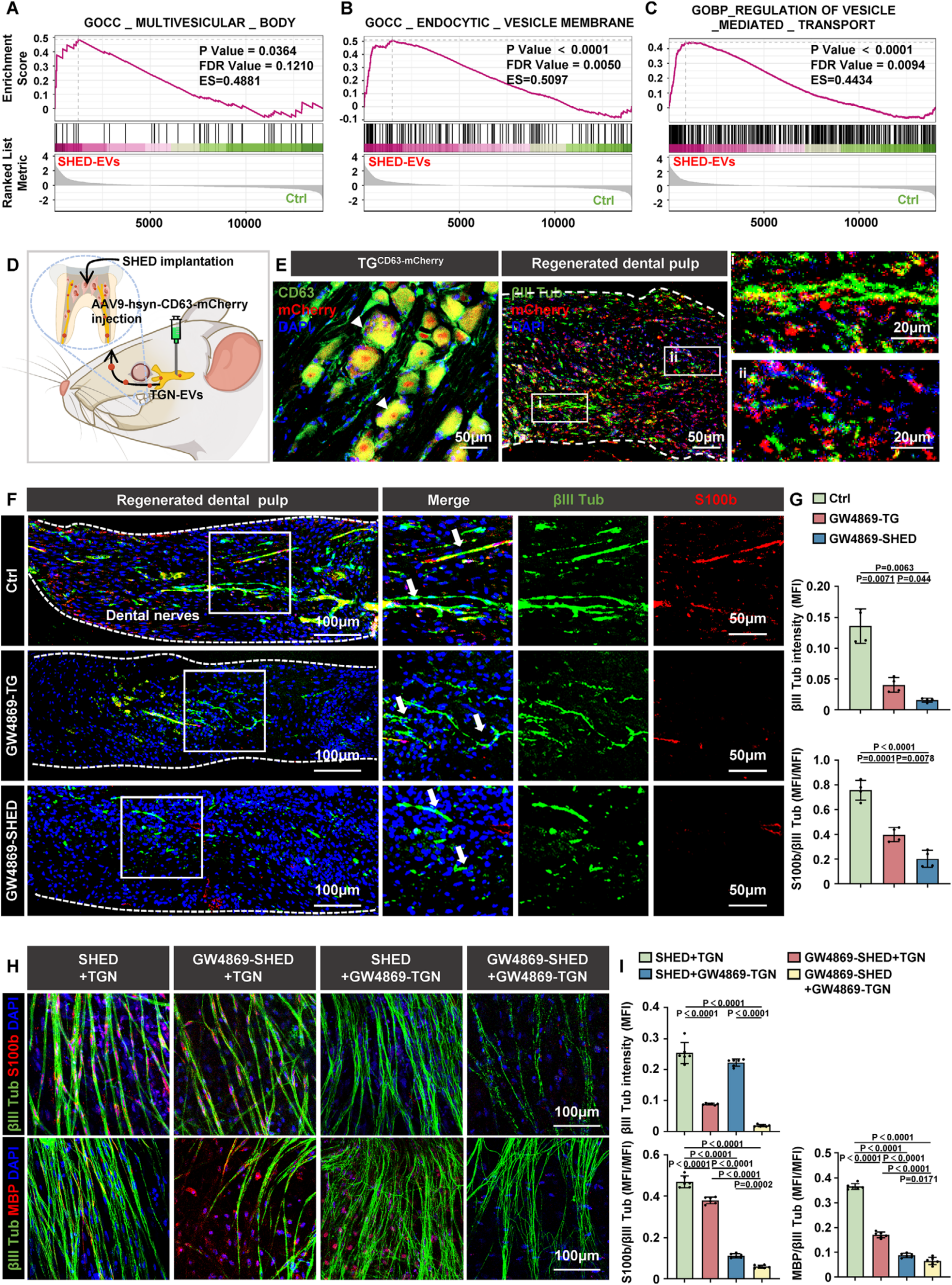
Reciprocal EVs communication between donor SHED and host TG in dental nerve regeneration. (A to C) Gene set enrichment analysis (GSEA) showing the significantly enriched pathways, including multivesicular body, endocytic vesicle membrane and regulation of vesicle mediated transport in TGN engulfed SHED‐EVs. (D) Schematic drawings of stereotaxic injection of AAV9‐hsyn‐CD63‐mCherry after SHED implantation in rats. (E) Immunostaining of in vivo tracing TGN‐EVs during dental pulp regeneration. Left panel shows the co‐localization of CD63 (green) and mCherry (red) in TG and white triangles indicate TGN. Middle panel shows the regenerated dental pulp and white boxes show the regenerated dental nerves (i) and mCherry labeled TGN‐EVs (ii) in dental pulp. Scale bar, 50 µm in left and middle panels and scale bar, 25 µm in right panel. (F and G) Immunostaining (F) and quantification (G) for regenerated axons and SC differentiation in regenerated rat dental pulps after 6 weeks SHED (Control group, Ctrl) or GW4869 treated SHED (GW4869‐SHED) implantation. GW4869 were injected into TG to inhibit EVs secretion (GW4869‐TG) with SHED implantation. Scale bar, 10 µm. Error bars denote SD from four regenerated rat dental pulps per condition. Statistics by Brown‐Forsythe and Welch ANOVA for βIII Tub intensity and one‐way ANOVA with Tukey's multiple comparison analysis for S100b/βIII Tub. (H and I) Immunostaining (H) and quantification (I) of regenerated axons and SC differentiation, as identified by S100b and MBP (myelin basic protein) in direct co‐culture of TGN and SHED for 6 weeks. TGN and SHED were respectively treated by GW4869 to inhibit EVs secretion before co‐culture. Scale bar, 100 µm. Error bars denote SD from six images per condition over three independent experiments. Statistics by one‐way ANOVA with Tukey's multiple comparison analysis for βIII Tub intensity, S100b/βIII Tub and MBP/βIII Tub.

To further validate the function of reciprocal EVs communication between SHED and TGN in rat dental nerve regeneration, GW4869 was injected into TG (GW4869‐TG group) through the infraorbital foramen as described by the previous study [36] or pretreated with SHED (GW4869‐SHED group) for the implantation. In the control group, the regenerated axons were enwrapped by SCs after SHED implantation. In the GW4869‐TG group, the dental nerves still could innervate the regenerated dental pulp, however, the SC differentiation was significantly inhibited, indicating EVs from host TG regulated SC differentiation of donor SHED in dental nerve regeneration (Figure 4F,G). Meanwhile, axons and SCs were rarely appeared in regenerated dental pulp of GW4869‐SHED implantation group (Figure 4F,G), indicating axonal regeneration is a prerequisite for inducing SC differentiation of SHED.

To more directly explore the function of reciprocal EVs communication between SHED and TGN, we established an in vitro co‐culture model of these two cell types. SHED and TGN were pretreated with GW4869 respectively, and co‐cultured directly to investigate the role of reciprocal EVs communication between them. The axonal density was decreased in the GW4869‐SHED + TGN group. The SC differentiated markers (S100b and MBP) were decreased significantly in SHED + GW4869‐TGN group as expected, whereas both axonal density and SC differentiation were inhibited in GW4869‐TGN + GW4869‐SHED group (Figure 4H,I). Furthermore, shRAB27A was respectively transfected into SHED (shRBA27A‐SHED) and shRab27a into TGN (shRab27a‐TGN) to suppress the secretion of EVs more precisely and efficiently. shRNA control was transfected as a negative control (NC) group. Consistent results were obtained after co‐culture of SHED and TGN for 6 weeks in different treatment groups (Figure S6E,F). Collectively, our findings indicate that reciprocal EVs communication between donor SHED and host TG is critical for dental pulp nerve regeneration, as inhibiting SHED‐EVs secretion decreases axonal regeneration and inhibiting TG‐EVs reduces SC differentiation of implanted SHED.

### 2.5 EVs From TGN After Internalization of SHED‐EVs Promote the Differentiation of SHED Into SCs In Vitro

Further, we isolated and characterized rat TGN‐EVs, which showed bilayer membrane spheres, with an average diameter equaled to 135.4 nm and expressed with the EVs markers CD9, CD63, TSG101 and Alix and negative for Calnexin (Figure 5A, B and Figure S7A). SHED and rat TGN were cultured on two separate glass slides in one petri dish to verify the reciprocal EVs traffic. Similar amounts of PKH67‐labeled TGN‐EVs and PKH26‐labeled SHED‐EVs were mixed and placed in the petri dish (Figure 5C). After 24 h of incubation, SHED preferred to endocytose more TGN‐EVs than SHED‐EVs (the ratio of engulfed TGN‐EVs/ SHED‐EVs in SHED was 1.86 ± 0.31), while TGN endocytosed more SHED‐EVs than TGN‐EVs (the ratio of engulfed TGN‐EVs/ SHED‐EVs in TGN was 0.47 ± 0.12) (Figure 5D,E). The endocytic preference of SHED and TGN towards each EVs was also independently evaluated. The uptake of TGN‐EVs by SHED was higher than that by TGN (the ratio of engulfed TGN‐EVs in SHED to TGN is 3.90 ± 1.66) (Figure S7B,C,E), while the uptake of SHED‐EVs by TGN was higher than that by SHED (the ratio of engulfed SHED‐EVs in TGN to SHED was 1.86 ± 0.32) (Figure S7,B,D,F). These findings demonstrate that SHED and TGN have preferential engulfment of EVs derived from other cells, thereby providing further support for the utility of our in vitro co‐culture model in investigating reciprocal EVs interplay.

**FIGURE 5:**
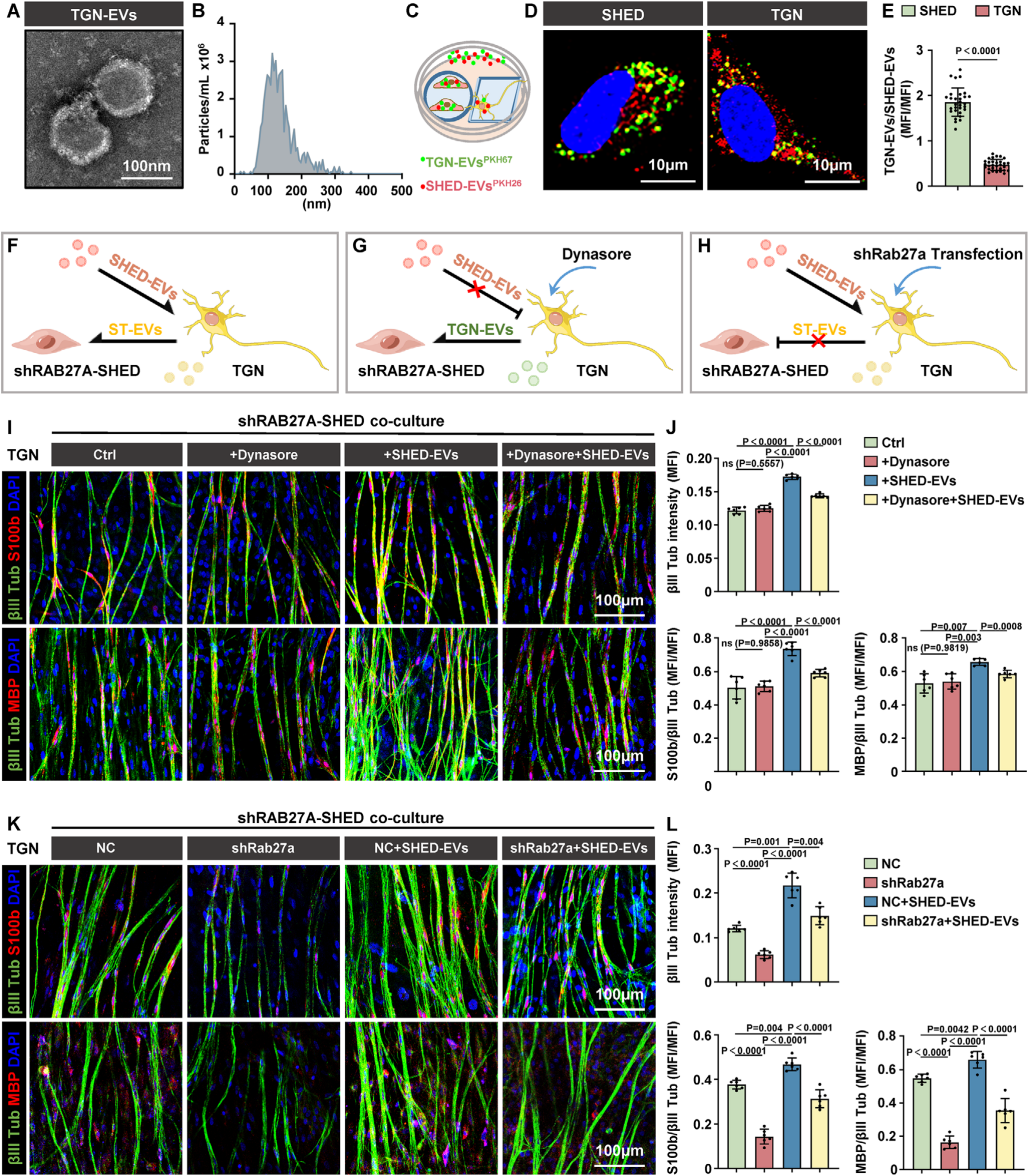
EVs from TGN after internalization of SHED‐EVs promote the differentiation of SHED into SCs in vitro. (A and B) The characterization of TGN‐EVs, TEM analysis of TGN‐EVs (A) and NTA analysis of TGN‐EVs (B). Scale bar, 100 nm. (C to E) Schematic drawings to compare preferential engulfment of EVs between SHED and TGN (C). Internalization of PKH67 labeled TGN‐EVs (green) and PKH26 labeled SHED‐EVs (red) in SHED and TGN (D). Scale bar, 10 µm. Quantification of internalized TGN‐EVs /SHED‐EVs mean fluorescence intensity ratio (green/red) in SHED and TGN (E). Error bars denote SD from thirty SHED and TGN. Statistics by Welch's *t* test. (F to H) Schematic drawings of temporal sequence in reciprocal EVs traffic between SHED and TGN. shRAB27A‐SHED were used in co‐culture system with TGN to eliminate the interference caused by SHED‐EVs released during co‐culture process. (I and J) Immunostaining (I) and quantification (J) of regenerated axons and SC differentiation, as identified by S100b and MBP in direct co‐culture of SHED and TGN. DIV3 TGN were pretreated with dynasore to inhibit EVs internalization, while SHED‐EVs (30 µg/ml) were added into TGN. shRAB27A‐SHED were co‐cultured with pre‐treated TGN for 6 weeks. Scale bar, 100 µm. Error bars denote SD from six images per condition over three independent experiments. Statistics by one‐way ANOVA with Tukey's multiple comparison analysis for βIII Tub intensity and S100b/βIII Tub and Brown‐Forsythe and Welch ANOVA for MBP/βIII Tub. (K and L) Immunostaining (K) and quantification (L) of regenerated axons and SC differentiation, as identified by S100b and MBP in direct co‐culture of SHED and TGN. DIV3 TGN were transfected with shRab27a to inhibit EV secretion, while SHED‐EVs (30 µg/ml) were added into TGN. shRAB27A‐SHED were co‐cultured with transfected TGN for 6 weeks. Scale bar, 100 µm. Error bars denote SD from six images per condition over three independent experiments. Statistics by Brown‐Forsythe and Welch ANOVA for βIII Tub intensity and one‐way ANOVA with Tukey's multiple comparison analysis for S100b/βIII Tub and MBP/βIII Tub.

Exogenous EVs can be taken up by neurons and hijack the secretory endosomal pathway. They are then processed in endosomes and transmitted to downstream neurons, which permits long‐distance transportation via EVs [29]. Considering that the regeneration of axons relies on the engulfment of SHED‐EVs, we hypothesized that the reciprocal EVs interplay occurred in a sequential manner. Specifically, EVs derived from TGN after internalization of SHED‐EVs (ST‐EVs) promoted the differentiation of SHED into SCs. To verify that, a direct co‐culture model of shRAB27A‐SHED with TGN was established to eliminate the interference exerted by EVs release of SHED during the co‐culture process (Figure 5F–H). External SHED‐EVs were pre‐incubated with TGN and co‐cultured with shRAB27A‐SHED (+SHED‐EVs) (Figure 5F). TGN were pretreated with Dynasore, a dynamin inhibitor that blocks EVs uptake, and subsequently incubated with SHED‐EVs (+Dynasore+SHED‐EVs) (Figure 5G). The results demonstrated that the axonal regeneration of TGN and SC differentiation were significantly enhanced in shRAB27A‐SHED co‐cultured with TGN pre‐internalizing of SHED‐EVs (+SHED‐EVs) (Figure 5I,J). Conversely, inhibition of SHED‐EVs uptake in TGN by Dynasore (+Dynasore+SHED‐EVs) attenuated these processes (Figure 5I,J). Meanwhile, Dynasore treatment (+Dynasore) of TGN showed no effect on SC differentiation (Figure 5I,J). These results demonstrate that inhibition of SHED‐EVs uptake in TGN decreases the axonal growth and SC differentiation of SHED.

Next, the impact on SC differentiation was verified when inhibiting ST‐EVs secreted from TGN by shRab27a transfection (Figure 5H). When shRab27a‐SHED was co‐cultured with shRab27a‐TGN (shRab27a), the axonal regeneration and SC differentiation were notably decreased compared to co‐culture with negative control TGN (NC‐TGN) (Figure 5K,L). Moreover, supplementation of SHED‐EVs to NC‐TGN (NC+SHED‐EVs) significantly enhanced axonal regeneration and SC differentiation in the co‐culture model (Figure 5K,L). Nevertheless, these enhancements were attenuated when co‐cultured shRab27a‐SHED with shRab27a‐TGN supplemented with SHED‐EVs (shRab27a+SHED‐EVs) (Figure 5K,L). These results demonstrate that inhibiting EVs secretion of TGN, especially ST‐EVs, will decrease SC differentiation of SHED.

### 2.6 ST‐EVs Enriched With TUFM and GFM2 Promote the Differentiation of SHED Into SCs

The mechanism underlying SC differentiation of SHED promoted by ST‐EVs was further examined. ST‐EVs are presented as spherical structures with an average diameter of 122.6 nm (Figure S8A,B). After treatment with ST‐EVs, SHED exhibited a significantly more elongated shape compared to those treated with PBS, SHED‐EVs and TGN‐EVs groups (Figure 6A,B). The expression of Schwann related markers was significantly increased in ST‐EVs stimulated SHED compared to PBS, SHED‐EVs, and TGN‐EVs treatment groups (Figure 6C). Moreover, the co‐culture of TGN with SHED‐EVs (+SHED‐EVs), TGN‐EVs (+TGN‐EVs), and ST‐EVs (+ST‐EVs) treated SHED for 6 weeks showed that the axonal bundles were enwrapped by more SCs in the ST‐EVs group as demonstrated by significant increases in the expression levels of S100b and MBP expression (Figure 6D,F and Figure S8D,E). The co‐culture of TGN with different EVs treated SHED was implanted into empty root canals and transplanted subcutaneously into immunocompromised mice. More SCs were observed in the ST‐EVs group (Figure 6E,G and Figure S8F,G), which also possessed the most potent ability in inducing SC differentiation compared to te SHED‐EVs and TGN‐EVs groups.

**FIGURE 6:**
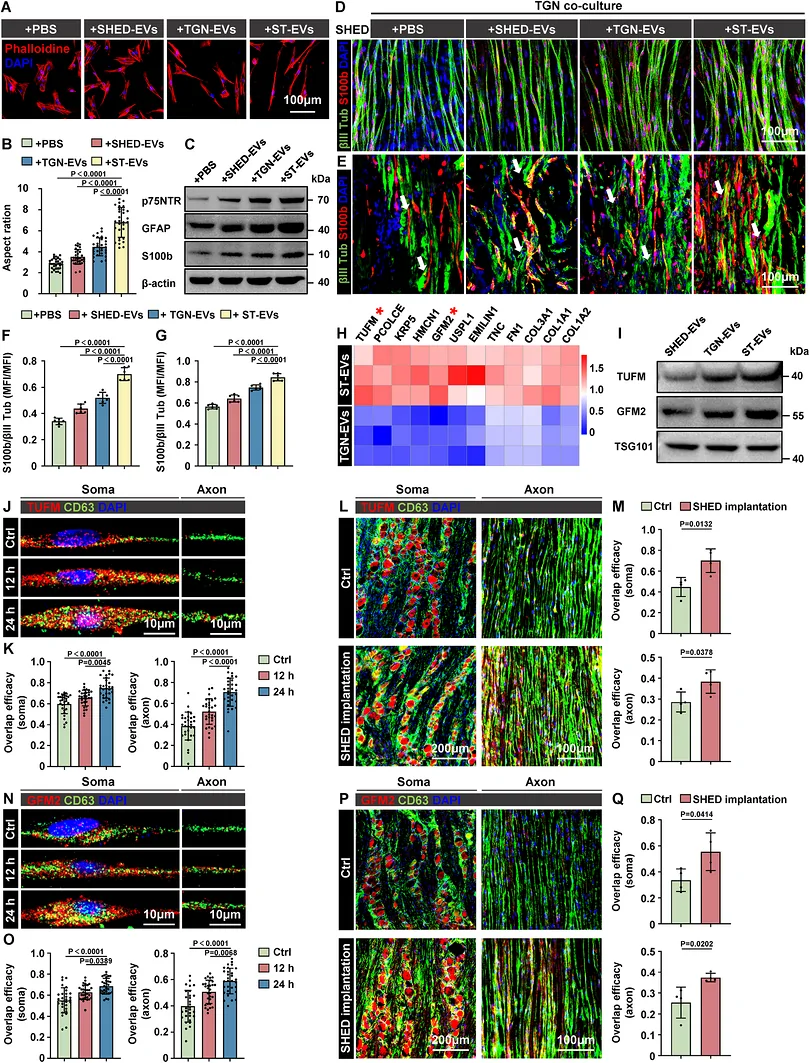
ST‐EVs enriched with TUFM and GFM2 promote the differentiation of SHED into SCs. (A to C) SC differentiation of SHED after stimulated by SHED‐EVs, TGN‐EVs and ST‐EVs at same concentration with 30 µg/ml. The morphology was detected by Phalloidine staining (A), analyzed with aspect ratio (B), and the SCs makers were measured by western blot (C). Scale bar, 200 µm. Error bars denote SD from thirty SHED per condition. Statistics by Brown‐Forsythe and Welch ANOVA. (D and F) Immunostaining (D) and quantification (F) of SC differentiation (S100b) in direct co‐culture of SHED and TGN. SHED were pre‐treated with SHED‐EVs, TGN‐EVs and ST‐EVs at same concentration with 30 µg ml^−1^ and co‐cultured with TGN for 6 weeks. Scale bar, 100 µm. Error bars denote SD from six images per condition over three independent experiments. Statistics by one‐way ANOVA with Tukey's multiple comparison analysis. (E and G) Immunostaining (E) and quantification (G) of SC differentiation (S100b) in co‐cultured SHED and TGN subcutaneously implanted into immunocompromised mice with empty root canal for 6 weeks. SHED were pre‐treated with SHED‐EVs, TGN‐EVs and ST‐EVs at same concentration with 30 µg/ml and co‐cultured with TGN for 1 week before implantation. Scale bar, 100 µm. Error bars denote SD from six immunocompromised mice per condition. Statistics by one‐way ANOVA with Tukey's multiple comparison analysis. (H) Heat maps showed the up‐regulated proteins in ST‐EVs compared to TGN‐EVs. Red asterisks denote Tufm and Gfm2. (I) The expression of TUFM and GFM2 in SHED‐EVs, TGN‐EVs and ST‐EVs by western blot. (J, K, N and O) Immunostaining (J and N) and quantification (K and O) of the co‐localization of TUFM or GFM2 with CD63 in soma and axon of TGN stimulated by SHED‐EVs for 12 h and 24 h. Scale bar, 10 µm. Error bars denote SD from thirty TGNs per condition. Statistics by one‐way ANOVA with Kruskal‐Wallis test for TUFM in soma and one‐way ANOVA with Tukey's multiple comparison analysis for other data. (L, M, P and Q) Immunostaining (L and P) and quantification (M and Q) of co‐localization of TUFM or GFM2 with CD63 in rat TG after implantation of SHED into maxillary first molars. Scale bar, 200 µm in soma and 100 µm in axon. Error bars denote SD from four rat TGs per condition. Statistics by unpaired t test.

A 4D‐label free proteomic analysis was performed to identify EVs proteins and elucidate the differences in content between TGN‐EVs and ST‐EVs. Among the 12 upregulated proteins (fold change > 1.5; *p*‐value < 0.05), the expression of two mitochondria‐located proteins, TUFM, and GFM2 (ribosome‐releasing factor 2, mitochondrial), was significantly increased (Figure 6H and Figure S8C). We focused on TUFM and GFM2 because EVs‐delivered mitochondrial proteins tune neuronal‐SCs metabolism [37], and such cargo can reprogram recipient‐cell fate [38, 39]. In addition, innervation demands mitochondrial transfer for energetic support [40], and both proteins are enriched in ST‐EVs and control mitochondrial biogenesis. These findings therefore warrant further investigation into the functional roles of TUFM and GFM2.The expression levels of TUFM and GFM2 were also examined by western blot, which were highest in ST‐EVs compared to SHED‐EVs and TGN‐EVs (Figure 6I). In addition, the colocalization of TUFM, GFM2, and TOMM20 puncta with the EVs marker CD63 in the soma and axon of TGN was measured after the SHED‐EVs stimulation. The increased colocalization of TUFM, GFM2, and TOMM20 with CD63 in TGN was observed in a time‐dependent manner, indicating their increased packaging into ST‐EVs (Figure 6J,K,N,O and Figure S8H,I).

Maxillary first molars with SHED implantation were used to examine the increased packaging of TUFM and GFM2 into ST‐EVs during dental pulp regeneration in vivo, while molars with pulpectomy were used as a control group. Interestingly, increased colocalization of TUFM, GFM2, and TOMM20 puncta with CD63 in TGN and axons of TG was observed in the SHED implantation group (Figure 6L,M,P,Q and Figure S8J,K). In conclusion, these results confirm that the upregulation of TUFM and GFM2 in ST‐EVs is released by TGN after internalization of SHED‐EVs, which leads to increased SC differentiation of SHED.

### 2.7 ST‐EVs Transfer TUFM to Promote SC Differentiation of SHED

To further confirm the functions of delivering TUFM and GFM2 proteins to human cells by ST‐EVs due to their rat‐specific nature, we compared the amino acid sequence and found a 93.81% similarity between rat Tufm and human TUFM and an 82.53% similarity between rat Gfm2 and human GFM2 (Table S1). The structures of these proteins were predicted using a template‐based AlphaFoldDB modeling approach [41, 42]. Intriguingly, the N‐terminal extension structure, resembling a mitochondrial targeting sequence, exhibited similarities between the human and rat TUFM, but displayed inversion in GFM2 (Figure S9A). To determine whether ST‐EVs transfers TUFM and GFM2 to regulate SC differentiation, we transfected TGN with lentivirus to successfully downregulate the content of TUFM and GFM2 in ST‐EVs^shTufm^ and ST‐EVs^shGfm2^, respectively (Figure S9B). The addition of ST‐EVs^shTufm^ did not result in elongation of SHED or an increase in the expression of Schwann‐related markers in SHED, while the addition of ST‐EVs and ST‐EVs^shGfm2^ induced SC differentiation of SHED (Figure 7A–C). These results prove that TUFM in ST‐Exo is crucial for inducing SC differentiation of SHED.

**FIGURE 7:**
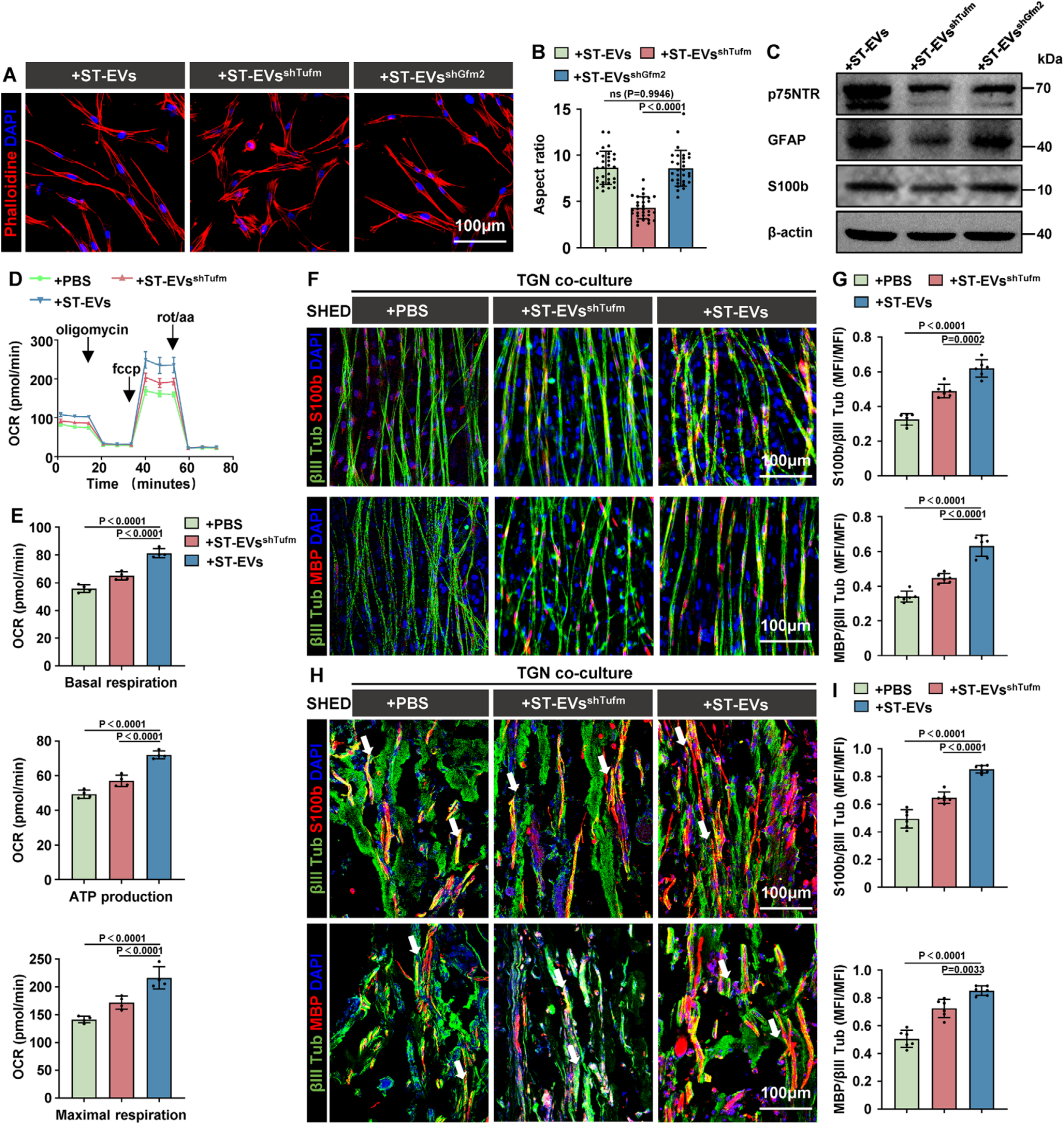
ST‐EVs transfer TUFM to promote SC differentiation of SHED. (A to C) SC differentiation of SHED after stimulated by ST‐EVs, ST‐EVs^shTufm^ and ST‐EVs^shGfm2^ at same concentration of 30 µg ml^−1^. The morphology was detected by Phalloidine staining (A), analyzed with aspect ratio (B), and the SCs‐related makers were measured by western blot (C). Scale bar, 200 µm. Error bars denote SD from thirty SHED per condition. Statistics by one‐way ANOVA with Tukey's multiple comparison analysis. (D and E) OCR traces during Seahorse extracellular flux experiment (D). Quantification of basal respiration, ATP production and maximal respiration (E). Error bars denote SD from four independent samples per condition. Statistics by one‐way ANOVA with Tukey's multiple comparison analysis. (F and G) Immunostaining (F) and quantification (G) of SC differentiation as identified by S100b and MBP in direct co‐culture of SHED and TGN. SHED were pretreated with PBS, ST‐EVs^shTufm^ or ST‐EVs at same concentration of 30 µg ml^−1^ and co‐cultured with TGN for 6 weeks. Scale bar, 100 µm. Error bars denote SD from six images per condition over three independent experiments. Statistics by one‐way ANOVA with Tukey's multiple comparison analysis. (H and I) Immunostaining (H) and quantification (I) of SC differentiation as identified by S100b and MBP in co‐cultured SHED and TGN after subcutaneously implanted into immunocompromised mice for 6 weeks. SHED were pre‐treated with PBS, ST‐EVs^shTufm^ or ST‐EVs at same concentration of 30 µg ml^−1^ and co‐cultured with TGN for 1 week before implantation. Scale bar, 100 µm. Error bars denote SD from six immunocompromised mice per condition. Statistics by one‐way ANOVA with Tukey's multiple comparison analysis.

TUFM is a highly conserved GTPase and responsible for mitochondrial protein translation, the pathogenic variant of which leads to oxidative phosphorylation (OXPHOS) deficiency [43]. Seahorse extracellular flux analysis was performed to monitor oxygen consumption rate (OCR) of SHED supplemented with ST‐EVs^shTufm^ or ST‐EVs. ST‐EVs significantly enhanced OXPHOS, including the basal respiration, ATP production, and maximal respiration compared to ST‐EVs^shTufm^ treated SHED (Figure 7D,E). Meanwhile, ST‐EVs^shTufm^ or ST‐EVs treated SHED were co‐cultured with TGN and ST‐EVs treatment showed significantly enhanced SC differentiation compared to ST‐EVs^shTufm^ (Figure 7F,G). After transplantation into immunocompromised mice, ST‐EVs exhibited increased SCs enwrapped with TGN compared to ST‐EVs^shTufm^ (Figure 7H,I). In sum, these data confirm the critical role of TUFM delivered by ST‐EVs in enhancing SC differentiation of SHED.

To further validate that the SC differentiation of SHED can be directly regulated by TUFM, the expression of TUFM in SHED was downregulated by shTUFM transfection (shTUFM‐SHED). Downregulation of TUFM inhibited OXPHOS in shTUFM‐SHED, and enhanced OCR (basal respiration, ATP production and maximal respiration) was obtained after stimulation with ST‐EVs compared to ST‐EVs^shTufm^ treatment (Figure S10A,B). Furthermore, the effect of TUFM downregulation in SHED on SC differentiation was also evaluated. In shTUFM‐SHED, the cellular morphology was shrunk and the expression of Schwann related markers was reduced compared to NC‐SHED, however, the morphology and Schwann related markers expression of which were rescued by ST‐EVs treatment (Figure S10C–E). Co‐culture of TGN with shTUFM‐SHED revealed decreased SC differentiation compared to with NC‐SHED, which was rescued in shTUFM‐SHED+ST‐EVs groups (Figure S10F,H). The shTUFM SHED co‐cultured with TGN were also subcutaneously transplanted into immunocompromised mice, the results of which are consistent with outcomes of the in vitro direct co‐culture (Figure S10G,I). Additionally, we also transfected lentivirus for overexpressing TUFM in SHED (OE TUFM‐SHED), which increased the SC‐related markers expression and promoted SC differentiation by co‐culturing with TGN (Figure S10J–L). Hence, our findings prove that the expression of TUFM in SHED is crucial for regulating the fate of SC differentiation.

## 3 Discussion

Donor cells implantation has successfully regenerated various tissues, yet the mechanisms underlying the integration of donor cells with host tissues for functional tissue regeneration remain unclear due to challenges in identifying implanted cells and host tissues [7]. In this study, we identified the fate of implanted SHED in pulp regeneration, which differentiated into SCs. Mechanistically, we found that reciprocal EVs communication between donor‐derived SHED and host TG regulated dental nerve regeneration. EVs derived from SHED enhanced axonal growth in the TG and stimulated TGN to secrete TUFM‐enriched EVs, which promoted the differentiation of SHED into SCs (Figure 8). Our study not only achieves chimeric regeneration by heterologous stem cells transplantation but also sheds light on the unrecognized intercellular mechanism that coordinates the integration of donor cells with host cells during tissue regeneration. Specifically, we propose that co‐culturing MSCs with TGN is a promising model to decipher the regulatory role of neurons and fate decision of MSCs during tissue regeneration.

**FIGURE 8:**
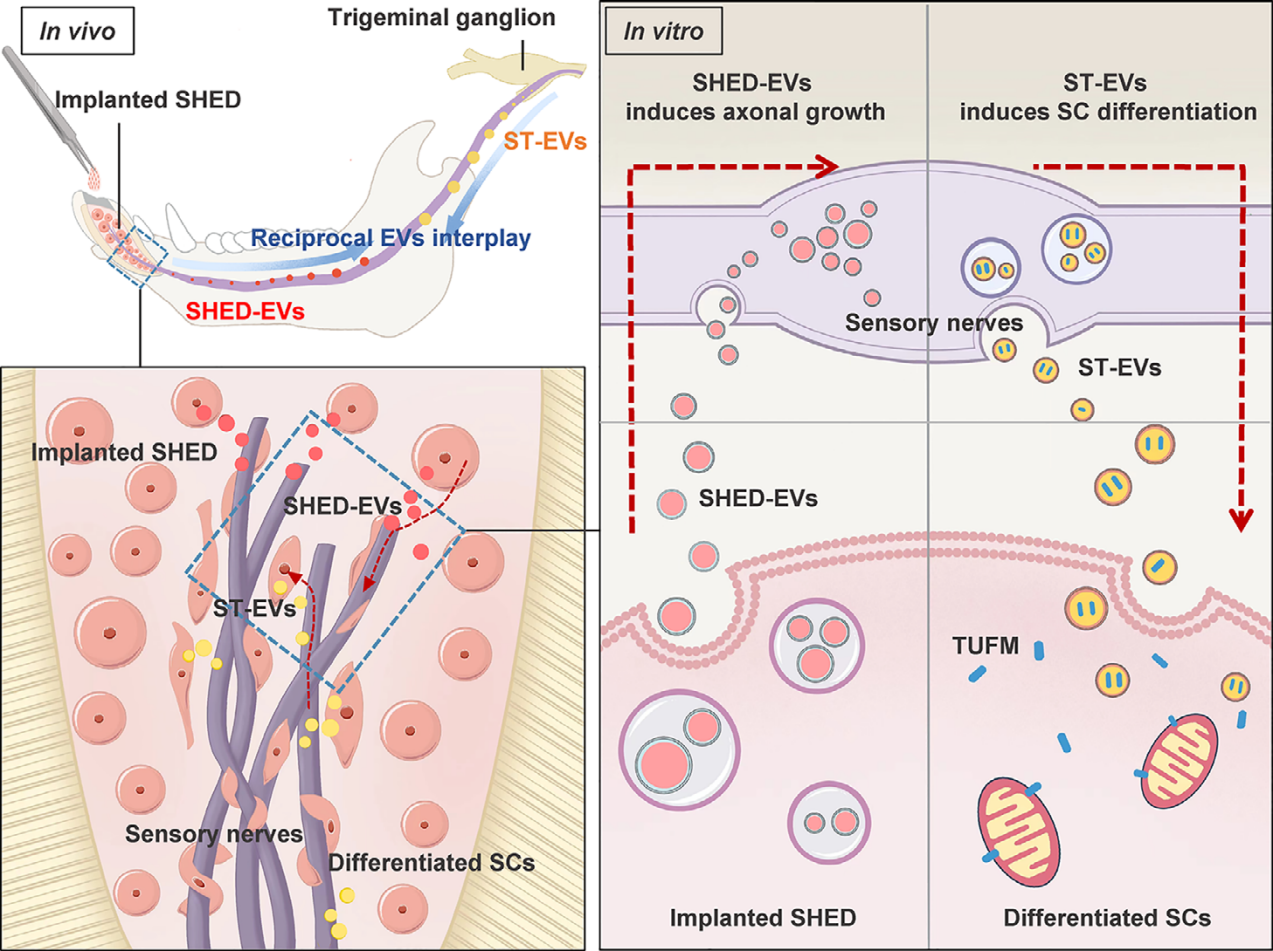
Implanted SHED integrate with host trigeminal axons to regenerate dental sensory nerves. Schematic illustration demonstrates that implanted SHED and host TG cooperate to regenerated dental pulp nerves via reciprocal EVs exchange. Mechanistically, SHED‐EVs migrate to TGN, promoting axonal growth. After engulfing SHED‐EVs, TG secreted ST‐EVs enriched with TUFM, which are subsequently delivered into SHED, thereby inducing SC differentiation of SHED.

The integration of implanted stem cells with host tissues is crucial for functional tissue regeneration, such as the integration of precursor cells into host retina photoreceptors to restore vision [8, 9, 44, 45]. However, the cellular communication mechanisms underlying the integration of donor and host cells during the regeneration process are limited by current models. In our previous study, we demonstrated that implanted donor tooth stem cells differentiate into odontoblasts for dentin formation, while host neurovascular bundles supply blood during syngeneic dental pulp regeneration [34, 46]. In this study, we have successfully regenerated human‐pig and human‐rat dental pulp using SHED transplantation. scRNA‐seq revealed that the regenerated dental pulp was integrated with donor stem cells and host derived endogenous cells, as implanted SHED differentiating into various functional cells, particularly SCs. Additionally, the supporting components like the neovascular bundle and immune cells were recruited from host. Our experimental models demonstrated that the regenerated dental nerves were composed of integrated endogenous axons and donor cells derived SCs. The chimeric human‐pig and human‐rat pulp regeneration models possess significant advantage for studying the integration of donor‐derived cells with host endogenous cells and exploring the cellular communication mechanism.

During tissue regeneration, implanted donor cells replenish defective tissues and recruit host components through paracrine signaling [11, 12]. Conversely, host‐derived neurovascular bundles provide a regenerative niche for progenitor cell [47], suggesting that host‐specific signals may influence the fate decisions of implanted cells. Recent studies have found that during tissue development and regeneration, the cell communication mediated by EVs was bidirectional among diverse cell types. Tooth germ epithelium and MSCs mutually exchange exosomes that promote amelogenesis and dentin mineralization, respectively, likewise, EVs communication between muscle and adipose stem cells drives myogenesis and suppresses adipogenesis [48, 49]. In this study, we have found that EVs derived from SHED induced innervation from TG during regenerated pulp formation. Functionally, SHED‐EVs were engulfed by TGN to promote axonal regeneration. Subsequently, TGN‐EVs retrograded back to implanted SHED and induced SC differentiation during dental pulp regeneration. Notably, both SHED‐EVs and TGN‐EVs showed a preference for reciprocal cell uptake rather than self‐uptake. The exosomal endocytic process is a highly specific process including clathrin/caveolae‐mediated endocytosis, phagocytosis, and micropinocytosis [50]. During tooth germ development, MSCs endocytose epithelial exosomes via clathrin and micropinocytosis, whereas, the process of MSCs‐exosomes endocytosed by epithelial cells is mediated by caveolae‐dependent endocytosis [49]. Nevertheless, dental pulp stem cells endocytose self‐secreted exosomes mainly by lipid raft/caveolar mediated endocytosis [51]. Hence, the preferential EVs internalization may be decided by the heterogeneity of donor/recipient cells and their EVs for specific functions. We also proved that temporal sequence existed in EVs communication between the host TGN and implanted SHED. As external signals have been shown to activate regenerative programs by altering genomic expression or EVs content of neurons [52, 53], sequential EVs communications also enhance the capacity of TGN to induce SC differentiation of implanted cells.

EVs can deliver mitochondrial proteins or mtDNA, activating OXPHOS and enhancing the multi‐differentiation ability of dental tooth stem cells [39, 54]. Injured axons release damage‐associated molecular patterns from mitochondria, which induce the migration of SCs [55]. We discovered that ST‐EVs carried significantly more TUFM and GFM2 compared to TGN‐EVs. Upon comparing the sequence and predicted structure of TUFM and GFM2 between humans and rats, we found a high degree of homology in TUFM, whereas GFM2 showed less homology. TUFM, an elongation factor involved in regulating the translational expression of mitochondrial DNA, plays an indispensable role in regulating OXPHOS. Activation of OXPHOS maintains the regenerative capabilities of stem cells [56, 57]. Downregulating the expression of TUFM inhibited the SC differentiation of SHED in this study. However, by introducing ST‐EVs to the cells, the TUFM carried by these EVs rescued SC differention of SHED with downregulation of TUFM. Thus, dental pulp regeneration is a highly collaborative process that involves not only the cellular integration of SHED and host TG but also the utilization of specific signals delivered through EVs communication.

In peripheral nerve injury, SCs migrate and proliferate to form Büngner bands, initiating the regeneration of the proximal stump of nerves [58]. This partnership between axon outgrowth and SC migration is intimate and mutually reinforced [59]. However, our findings indicate that, during dental pulp regeneration, nerve ingrowth is earlier than SCs differentiation. This could be attributed to the fact that nerves grow directly within the MSC scaffolds rich in abundant extracellular matrix (ECM), while SC differentiation is a comparatively slower process. In normal dental pulp, more than three quarters of SCs are non‐myelinated and associate with sensory nerve terminals through the odontoblast layer, participating in reparative dentin formation [14]. During mouse dental development, a significant population of MSCs in the tooth germ originates from peripheral nerve‐associated SCs, which differentiate into dental stem cells and odontoblasts [15, 60]. In our study, we observed a significant proportion of MSCs differentiating into SCs. We hypothesize that sensory innervation may recapitulate the tooth developmental process, thereby influencing the fate decision of implanted SHED. However, the mechanisms by which these differentiated SCs contribute to dental pulp regeneration still require further investigation.

Several limitations inherent to this study warrant further exploration. First, while our research group has previously demonstrated that autologous SHED transplantation can achieve functional dental pulp regeneration in young permanent teeth and restore sensory function through two‐year follow‐up preclinical experiments. However, limited by the chosen pulp regeneration model, this study lacks evaluation of sensory nerve function following xenogeneic SHED transplantation in miniature pigs and rats. Second, although we observed temporal sequence in axonal regeneration, SC differentiation and EVs mediated communication in ectopic models, these phenomena should be further validated in the orthotopic transplantation model. Finally, although SHED has been shown to possess low immunogenicity and no tumorigenic potential, immune related detections should be assessed during xenotransplantation.

In summary, our study demonstrates that the host TG coordinates with heterologously implanted SHED to regenerate functional dental pulp through reciprocal EVs interplay. EVs derived from implanted stem cells migrate to the host TG to activate axonal regeneration. As a feedback mechanism, EVs derived from the host TG, after internalizing SHED‐EVs, return to the donor cells carrying a higher concentration of TUFM, thereby inducing SC differentiation. Our findings highlight that reciprocal EVs interplay between host and donor cells coordinates both exogenous and endogenous integration, contributing to the achievement of functional tissue regeneration.

## Author Contributions

Conceptualization: B.L. and Y.J. Methodology: W.Z., J.G., J.D. and W.W. Investigation: W.Z., W.W., J.L. D.T. and J.D. Visualization: W.Z., J.G., W.W. and J.L. Funding acquisition: B.L. S.L. and Y.J. Project administration: B.L. Supervision: B.L. and Y.J. Writing – original draft: W.Z., J.G., W.W., S.L. and B.L. Writing – review and editing: B.L., Y.J., T.C., Z.L. and S.S.

## Ethics Statement

All animal experiments were approved by the Animal Care Committee of the Fourth Military Medical University and performed in accordance with the guidelines of the Institutional Animal Care Use Committee of the Fourth Military Medical University (20250009). The use of human deciduous tooth stem cells (SHED) were approved by the Ethics Committee of the Fourth Military Medical University and obtained informed consent from the parental/legal guardian (IRB‐REV‐2015034).

## Conflicts of Interest

The authors declare no conflicts ofg interest.

## Supporting information




**Supporting File**: exp270229‐sup‐0001‐SuppMat.zip

## Data Availability

All data are available in the main text or the supplementary materials. The study used commercially available antibodies and reagents, with details provided in tables S2. The raw sequence data of scRNA‐seq reported in this paper have been deposited in the GSA in National Genomics Data Center, China National Center for Bioinformation / Beijing Institute of Genomics, Chinese Academy of Sciences (GSA‐Human: HRA006547) that are publicly accessible at https://ngdc.cncb.ac.cn/gsa‐human. The mass spectrometry proteomics data have been deposited to the ProteomeXchange Consortium (https://proteomecentral.proteomexchange.org) via the iProX partner repository with the dataset identifier PXD048746. The RNA‐Seq data were deposited in the NCBI’ s Sequence Read Archive (SRA) (https://www.ncbi.nlm.nih.gov/) under accession number PRJNA1175018.
