# Supplementary material for: Endogenous Trigeminal Neurons Induce Schwann Differentiation of Donor Stem Cells in Dental Pulp Regeneration via Reciprocal Extracellular Vesicles Interplay
Source: Exploration (Beijing). 2026 Oct 8;6(5):70229. doi: 10.1002/exp2.70229 (PMC13650028; doi:10.1002/exp2.70229)
Supplement: Supplementary file 1 [file EXP2-6-70229-s001.zip › exp270229-sup-0001-SuppMat-verified/exp270229-sup-0001-SupMat.docx]

Endogenous Trigeminal Neurons Induce Schwann Differentiation of Donor Stem Cells in Dental Pulp Regeneration via Reciprocal Extracellular Vesicles Interplay

**List of Supplementary Materials**

MATERIALS AND METHODS

Figure. S1. SHED implantation regenerates dental pulp with SCs enwrapped nerves.

Figure. S2. Representative featured gene expression in SCs, MSCs, DPCs and endothelium.

Figure. S3. Implanted SHED integrate with host cellular component to regenerate dental pulp.

Figure. S4. SHED-EVs trigger host axonal growth and dental nerve regeneration.

Figure. S5. SHED-EVs trigger dental nerve regeneration in rats.

Figure. S6. Reciprocal EVs communication between donor SHED and host TG in dental nerve regeneration.

Figure. S7. EVs from TGN after internalization of SHED-EVs promote the differentiation of SHED into SCs in vitro.

Figure. S8. ST-EVs enriched with TUFM and GFM2 promote the differentiation of SHED into SCs.

Figure. S9. The homology of TUFM and GFM2 in human and rat.

Figure. S10. ST-EVs transfer TUFM to promote SC differentiation of SHED.

Table S1 The similarity of protein sequence of TUFM and GFM2 in human and rat

Table S2 Reagents and resources

**MATERIALS AND METHODS**

**Animals**

All Sprague Dawley (SD) rats (female, aged 8-10 weeks) and immunocompromised mice (male, aged 4-5 weeks) were purchased from The Fourth Military Medical University (FMMU) and maintained at room temperature (22-25℃) with 12 h light/dark cycle. The animals were free access to food and water. Minipigs (female, aged 18 months) were purchased from Shichuang Technology miniature Pig Breeding Base (Beijing, China). SD rats at postnatal day 3 of both sexes were used for primary trigeminal ganglion neurons (TGN) culture. All animal experiments were approved by the Animal Care Committee of the FMMU and performed in accordance with the guidelines of the Institutional Animal Care Use Committee of the FMMU (20250009).

**Antibodies and reagents**

All antibodies and chemical reagents and other resources used in this study are listed in table S2.

**Primary cells culture**

The use of human deciduous tooth stem cells (SHED) were approved by the Ethics Committee of the School of Stomatology, Fourth Military Medical University and obtained informed consent from the parental/legal guardian (IRB-REV-2015034). The SHED primary culture was performed in accordance with the ethical standards as laid down in the 1964 Declaration of Helsinki and its later amendments or comparable ethical standards. Briefly, the deciduous pulp tissues were cut into 1mm^3^ pieces and digested with 3 mg/ml collagenase I (Gibco, 17018029) for 1 hour at 37 ℃. The digested single-cell suspension was transferred into a 25 cm^2^ flask (NUNC, 156367) at 37 ℃ with 5% carbon dioxide. The adherent cells were expanded in culture medium consisting of alpha-Minimum Essential Medium (α-MEM, Gibco, 2110309) with 10% fetal bovine serum (FBS, Gibco, 10099141C), 100 U/ml penicillin and 100 μg/ml streptomycin (Gibco, 15070063). After 2 weeks, the cells were expanded and showed normal morphology of MSCs. SHED aggregates were used for dental pulp implantation and cultured as previously described ^[61]^. Briefly, P4 SHED were digested with trypsin (Gibco, 25200056) and 5 × 10^5^ cells were seeded into one well of a six-well plate (NUNC, 140675). When the cells reached approximately 80% confluence, the culture medium was changed with new medium containing 50 μg/ml vitamin C (Sigma, A92902). The medium was refreshed every 2 days. After 12 days of incubation, a white membrane structure could be obtained for implantation.

Primary TGN culture was prepared as previously described ^[62]^. Trigeminal ganglion (TG) was isolated from neonatal SD rats after being anesthetized by intraperitoneal injection with pentobarbital sodium. TGs were exposed by removing the skull of head. The brain and blood vessels were removed by ophthalmic forceps. TGs were collected into a 35-mm petri dish with DMEM (Gibco, 31600083) and cut into 1mm^3^ pieces. The tissues were followed by enzymatic digestion with Collagenase Type IV (Gibco, 17104019) for 10 min and trypsin for 10 min at 37 ℃ with 5% carbon dioxide. The residual TG tissues were dispersed into homogeneous solution by mechanical dissociation and the digestion was terminated by DMEM with 10% FBS. The cell suspension was centrifuged and resuspended with Neurobasal medium (Gibco, 21103049) supplemented with 2% B27 (Gibco, A1486701) and 1% Glutamax (Gibco, 35050079), and cultured on poly-L-Lysine (PLL, Solarbio, P8140) coated petri dish in incubator at 37 °C with 5% carbon dioxide.

**In situ dental pulp regeneration models**

General anesthesia was performed on minipigs by intravenous injection of pentobarbital sodium (40 mg/kg). After the oral cavity was sterilized, the crown of teeth was removed and the dental pulp was removed as previous described ^[23]^. The root canal was cleaned, irrigated with sterile saline and dried. SHED aggregates were implanted into prepared canal and sealed with composite resin. The teeth were extracted at 3 and 6 months after SHED implantation and fixed in 4% paraformaldehyde (Solarbio, P1110) for 24 h at 4 ℃. The teeth were scanned by Micro-CT (Siemens Inveon Micro‐CT, Germany), and 3D images were generated using Mimics Research 19.0 software. Subsequently, the teeth were demineralized in 17% EDTA (ph7.4, MP Biomedicals, 0219482290) for 8 weeks for histological analysis.

The dental pulp regeneration was also performed in the maxillary first molar of SD rats by implantation of SHED aggregates. After 6 weeks of SHED implantation, the maxillary bone was harvested, fixed in 4% paraformaldehyde, and demineralized for 4 weeks for histological analysis.

**Isolation and characterization of EVs**

When SHED were confluent to 80%, non-EVs medium was changed and the conditioned medium was collected after 48 h. SHED-EVs were isolated by differential ultracentrifugation as previously described.^[35]^ In brief, the medium was centrifugated at 300 × g for 10 min to remove cells, 2000 × g for 20 min to remove cell debris and 16,000 × g for 30 min to remove microvesicles. The supernatant was ultracentrifugated at 100,000 × g for 70 min to pellet EVs. After being resuspended in PBS, the supernatant was filtered through a 0.22-μm membrane to remove extracellular vesicles larger than 200nm and ultracentrifugated again. All the centrifugation was performed at 4 ℃ and the final SHED-EVs were resuspended by PBS and stored at -80 ℃.

To isolate TGN-EVs, the medium of TGN was changed at 4 days in vitro (DIV4) and collected after 48 h. To isolate ST-EVs, SHED-EVs were added into TGN at DIV2 to boost axon growth. At DIV4, the medium of SHED-EVs stimulated TGN was changed and collected after 48 h. Both TGN-EVs and ST-EVs were isolated by the same procedures with SHED-EVs.

The morphology of EVs was observed by transmission electron microscopy (TEM) (HITACHI, H7500, Japan). The size of EVs was measured using Nanosight tracking analysis (NTA) with a NanoSight NS300 instrument. EVs markers (CD9, CD63 and TSG101) were identified by western blot assay.

**EVs labeling and cellular uptake**

SHED-EVs were labeled with PKH26 red Fluorescent Cell Linker Kit (Sigma, MINI26-1KT) and TGN-EVs were labeled with PKH67 green Fluorescent Cell Linker Kit (Sigma, MINI67-1KT) according to manufacturer’s protocol. To compare the endocytic preference, SHED and TGN were cultured on two separate slides within the same petri dish. The same amount of labeled SHED-EVs and TGN-EVs were mixed up and added into the dish. After 24 h, both SHED and TGN were washed by PBS three times and fixed by 4% paraformaldehyde. The engulfed EVs were detected by immunofluorescence staining.

**Lentivirus transfection**

When reached 70% confluence, SHED were transfected with the following lentivirus according to the manufacturer’s protocol (Genechem Co., China). For labeling SHED-EVs delivering during dental pulp regeneration, SHED were transfected with CD63-conjugated mCherry plasmid and TUFM plasmid by lentivirus. For inhibiting SHED-EVs secretion, RAB27A short-hairpin RNA (shRAB27A, cgGATCAGTTAAGTGAAGAAA) was transfected by lentivirus. For knockdown of TUFM, shTUFM (CCAGGTTTACATCCTCAGCAA) was transfected into SHED. TGN were transfected with shTufm (CCCTGGTCATGCAGATTAT) or shGfm2 (GGATCTGCTTTCTAAACAAGA) at DIV3 to knockdown the expression of TUFM and GFM2 in TGN-EVs. shRab27a (ACAAACATAAGCCAAGCAATT) was transfected to inhibit TGN-EVs secretion. For labeling Tufm in TGN-EVs, Tufm-conjugated GFP plasmid was transfected by lentivirus. Cells transfected with shRNA lentivirus were selected by puromycin (MedChemExpress, HY-B1743A, 2mg/L). The control group of shRNA knockdown was transfected with negative control shRNA and the sequences were provided by Genechem Co., China.

**Fluorescence tracing of SHED-EVs in vivo**

Rat pulps of the maxillary first molar were removed for ex vivo fluorescence tracing of SHED-EVs. Subsequently, 10 μg/ul DiR (Invitrogen, D12731)-labeled SHED-EVs were injected into pulp cavities. The PBS diluted SHED-EVs were injected into contralateral tooth as control group. The rats were then euthanized at 3 and 5 days after injection. Both maxillary bone and TGs were harvested and imaged by IVIS Spectrum In Vivo Imaging System (PerkinElmer, USA). The intensity of fluorescence signals was quantified by Living Image Software (Caliper LifeSciences, USA). For accurately tracing the SHED-EVs in TG, SHED-EVs were labelled with PKH26 and injected into rat pulp cavities after pulpectomy. The TGs were harvested 3 days and 5 days after injection and fixed for immunofluorescence staining. Moreover, CD63-mcherry SHED were implanted into rat pulp cavities after pulpectomy for tracing SHED-EVs during dental pulp regeneration. After 4 weeks of CD63-mCherruy SHED implantation, the TGs were harvested to detect the internalization of SHED-EVs, with mCherry signals specifically labeling SHED-EVs transported from dental pulp.

**TG injection**

Stereotactic microinjection was performed as previously described ^[63]^. Briefly, SD rats were anesthetized and fixed in a stereotaxic frame. The skull was drilled with a hole and a 10-μl syringe (Hamilton Company) was positioned at 2.5 mm posterior and 1.5 mm lateral to the bregma. The needle was advanced to the base of the skull with 0.1 mm retraction gently. Adeno-associated virus (AAV) with CD63-conjugated mCherry was constructed and purified according to standard protocol (Genechem Co., China). The neuron-affinity serotypes 9 was selected with neuron-specific promoter hsyn to specially express in neuron. The AAV was injected in a volume of 2ul over 10 minutes.

To inhibit EVs secretion of TG, GW4869 (10mM prepared in DMSO and diluted to 10μM in PBS, MedChemExpress, HY-19363) was injected into TG by a percutaneous approach via infraorbital foramen, which was used as previously described ^[36]^. After inserting into infraorbital foramen, the needle was advanced 22mm along infraorbital foramen to reach TG. GW4869 was injected in a volume of 20ul over 10 minutes. The GW4869 was injected every 3 days and DMSO diluted in PBS was used as control. After 2 weeks, all rats were used for dental pulp regeneration experiments and were euthanized 6 weeks after SHED implantation.

**Direct co-culture model of TGN and SHED**

The direct co-culture was performed as previously described ^[64]^. According to the experimental design, SHED and TGN were plated in two different schemes. In scheme 1, SHED (2 × 10^5^) were initially seeded in a 12-well plate and cultured with a-MEM containing 10% FBS and 50 μg/ml vitamin C for 7 days. Subsequently, TGN (5 × 10^5^) were introduced into the culture to establish co-culture condition. In scheme 2, TGN (5 × 10^5^) were firstly seeded in a PLL coated 12-well plate and cultured using Neurobasal medium supplemented with 2% B27 and 1% Glutamax for 7 days. SHED (2 × 10^5^) were then added into the TGN culture. During direct co-culture, the medium was replaced with a-MEM with 10% FBS and 50 μg/ml vitamin C, and two third of the media was changed every 3 days.

**Indirect co-culture conditions of TGN and SHED**

The 6-well inserts with 0.4 mm pore size filter (Corning, 3450) were used following the manufacturer’s instruction. For detecting the effect of SHED on axonal growth, 2 × 10^5^ SHED were seeded into the well inserts with a-MEM medium and 10% FBS or pretreated with GW4869 (10mM prepared in DMSO and diluted to 10uM in PBS). 5 × 10^5^ TGN were seeded into PLL coated 6-well plates with Neurobasal medium and co-cultured with SHED. The TGN were fixed after co-cultured for 24 h and immunofluorescence stained with βIII Tubulin (βIII Tub). The axonal length was measured by Neuron J Plugin in ImageJ.

**In vivo subcutaneous implantation**

The third molar teeth were harvested from the Department of Oral-Maxillofacial in the School of Stomatology, Fourth Military Medical University. The outer periodontal ligament tissues, cementum and inner dental pulp with partial dentin were removed. The dental root pieces were ultrasonic cleaned with deionized water and then exposed to 17% EDTA for 5 min. Finally, the root slices were maintained in PBS with penicillin and streptomycin and prepared as a scaffold for dental pulp regeneration. After co-culturing SHED and TGN for 1 week, they were subsequently loaded into the tooth scaffold and implanted subcutaneously into immunocompromised mice for 6 weeks.

**Immunofluorescence staining**

Frozen sections were prepared as 10 μm and permeated with 0.3% Triton X-100 (MP Biomedicals, 194854). After blocked with goat serum (Boster, AR0009) for 1 hour at room temperature, sections were incubated with the following primary antibodies over night at 4 ℃: Anti-p75NTR (Cell Signaling Technology, 8238), Anti-S100b (Abcam, ab52642), Anti-GFAP (Merckmillipore, AB5541), Anti-MBP Antibody (Cell Signaling Technology, 78896), Anti-TUFM (Abcam, ab173300), Anti-GFM2 (Huabio, ER1909-51), Anti-MBP (Cell Signaling Technology, 78896), Anti-CD63 (Proteintech, 67605-1-Ig), Anti-TOMM20 (Abcam, ab186735), Anti-GFP (Abcam, ab1218), Anti-βIII Tubulin (Abcam, ab78078), Anti-βIII Tubulin (Abcam, ab18207)， Anti-CD90 (Abcam, ab181469), Anti-mCherry (Abcam, ab167453), Anti-human mitochondria (Abcam, ab215892), Anti-CGRP (Cell Signaling Technology, 14959), Anti-TRPM8 (Proteintech, 12813-1-AP), Anti-TRPV1 (Abcam, ab305299), Anti-CD31 (Abcam, ab28364), or Anti-DSPP (Abcam, ab216892). After incubated in a dark and humidified chamber overnight at 4℃, the sections were washed three times in PBS and incubated with fluorescently conjugated secondary antibodies: FITC AffiniPure Goat Anti-Rabbit IgG (H+L) (Yeasen, 33107ES60), Cy™3 AffiniPure Goat Anti-Rabbit IgG (H+L) (Yeasen, 33108ES60), Alexa Flour ® 488 Goat Anti-Mouse IgG (Yeasen, 33206ES60), Alexa Flour ® 594Goat Anti-Mouse IgG (Yeasen, 33212ES60) and Alexa Fluor 647-conjugated secondary antibodies (Abcam, ab150171) in the dark chamber for 1 hour at room temperature. After washed three times by PBS, the nuclei were stained with DAPI (Yeasen, 40728ES03) for 10 min. Immunofluorescence images were obtained by confocal microscope (Nikon A1+, Japan).

**Cell morphological analysis**

After treated with different EVs for 24 h, SHED seeded on the coverslip were fixed with 4% paraformaldehyde for 10 min and incubated with 0.3% Triton X-100 for 20 min. After washed with PBS twice, SHED were stained with TRITC Phalloidin (Yeasen, 40734ES75, diluted with PBS into 100nM). The nuclei were counterstained with DAPI for 10 min. The aspect ratio of SHED was analyzed by ImageJ.

**Western blot**

Cells and EVs were lysed in RIPA (Beyotime, P0013B) supplemented with protease inhibitor (Roche, 04693132001) on ice immediately. The protein concentration of the lysate was determined by BCA protein assay kit and equal amounts of proteins were loaded on SDS-PAGE and transferred onto polyvinylidene fluoride (PVDF) membranes. The membranes were blocked in 5% non-fat powdered milk for 1hour at room temperature. Primary antibodies were used as follows: Anti-p75NTR (Cell Signaling Technology, 8238), Anti-S100b (Abcam, ab52642), Anti-GFAP (Cell Signaling Technology, 80788), Anti-TUFM (Abcam, ab173300), Anti-GFM2 (Huabio, ER1909-51), Anti-CD9 (Huabio, ET1601-9), Anti-CD63 (Proteintech, 67605-1-Ig), Anti-TSG101 (Abcam, ab125011), Anti-Alix (Abcam, ab186429), Anti-Calnexin (Huabio, ET1611-86) and Anti-beta actin (Proteintech, 81115-1-RR) at 4 ℃ overnight. The membranes were incubated by corresponding horseradish-peroxidase (HRP)-conjugated secondary antibody (Cell Signaling Technology, 7076 and 7074) for 1hour at room temperature. Afterward, the membranes were developed using the electrochemiluminescence (ECL) analysis (Tanon-Bio, 4600).

**Seahorse metabolic flux analysis**

SHED (1 × 10^5^/well) were seeded in Seahorse XFe96-cell culture plates (Agilent, USA) and treated with different EVs for 24 h. Prior to metabolic test, the medium was replaced with pre-warmed Seahorse XF assay medium (Agilent Technologies, #103575-100) and the SHED were cultured in CO_2_-free 37℃ incubator for 1 hour. Oxygen consumption rate (OCR) of SHED was performed by a Seahorse XFe96 Analyzer (Agilent, USA) according to the manufacturers protocol. In OCR assay, cells were measured at basal level and 1μM oligomycin, 2μM FCCP and 2μM rotenone/antimycin-A were sequentially injected. The data was analyzed using wave software (Wave desktop software, Agilent Technologies, China).

**Protein structure prediction**

The homology of TUFM and GFM2 in human and rat was compared by aligning amino sequence in Uniprot (<https://www.uniprot.org/>). The protein structure of human TUFM (P49411), rat TUFM (P85834) and human GFM2 (Q969S9) and rat GFM2 (Q5BJP6) was predicted by AlphaFold Protein Structure Database (<https://alphafold.ebi.ac.uk/>) ^[41-42]^.

**Detection of TUFM and GFM2 in intraluminal endosomal vesicles**

DIV2 primary TGN were pretreated with SHED-EVs (30 μg/ml) for 12 and 24hours and fixed respectively for immunofluorescence staining. Anti-CD63 (Proteintech, 67605-1-Ig) was used to label intraluminal endosomal vesicles. The co-localization of CD63 with TUFM and GFM2 in the soma and axon region of TGN were analyzed by Colocalization Finder in Image J respectively.

**Tissue dissociation and preparation of single-cell suspensions**

The regenerated dental pulps were extracted from minipig teeth at 6 months after implantation, washed 2-3 times in 4 ℃ precooled PBS on ice, then transferred to a sterile RNase-free culture dish and cut into small pieces about 0.5mm^2^. The samples were incubated in a shaker at 37℃ with the hydrolysate kit (Miltenyi Biotec, DS_130-095-929). Generally, the cell suspensions were centrifuged at 500r at 4℃ for 5min, discarded the supernatant and resuspended in PBS with 0.04% BSA. Then 40µm cell sieve was used to filter single-cell suspensions. Cell number and viability were assessed by the cell counter (Countstar Rigel S2, China). The concentration of suspension was adjusted to about 1 × 10^6^ /ml.

**Single-cell RNA sequencing (scRNA-seq)**

The single cell RNA-seq libraries were prepared with Chromium Next GEM Single Cell 3ʹ Reagent Kits v3.1 on the Chromium Controller (10 × Genomics). The cell suspensions were loaded onto the Chromium Next GEM Chip G and ran the Chromium Controller to generate single-cell gel beads in the emulsion (GEMs) according to the manufacturer’s recommendation. Captured cells were lysed and the released RNA was barcoded through reverse transcription in individual GEMs. Barcoded, full-length cDNA was generated and libraries were constructed according to the performer’s protocol. The quality of libraries was assessed by Qubit 4.0 and Agilent 2100. Sequencing was performed on the Illumina NovaSeq 6000 with a sequencing depth of at least 50,000 reads per cell and 150 bp paired-end reads (performed by Biomarker Technologies Corporation, Beijing, China). Alignment with the human genome (GRCh38) and pig genome (Sscrofa11.1) respectively, filtering, barcode counting, and UMI counting were performed with 10 ×CellRanger v7.0 to generate a feature-barcode matrix.

Overall, the numbers of actually sequenced cells were 22616 cells (9485 cells derived from implanted SHED and 13131 cells derived from host) in regenerated dental pulp at 3 months after SHED implantation, 28317 cells (10846 cells derived from implanted SHED and 17471 cells derived from host) in regenerated dental pulp at 6 months after SHED implantation and 9436 cells in normal dental pulp.

**Reduction, clustering, and identification of differentially expressed genes**

The feature-barcode matrix was subsequently processed using R v4.3.1 and Seurat v4.0 package ^[65]^. We applied the batch effect correction analysis by the Harmony package based on the top 2000 variable genes with the default harmony parameter ^[66]^. To remove the cells with low quality, cells with gene number over 300, the ratio of mitochondria lower than 0.10 and the ratio of ribosome lower than 0.2 were maintained, and genes with at least one feature count in more than three cells were used for the following analysis. The gene expression levels for each cell were normalized by NormalizeData function of Seurat. Cell cycle effects were regressed with ScaleData function of Seurat using cell cycle markers. The top 2,000 variable genes were selected by FindVariableFeatures for principal component analysis. Cells were grouped into clusters by FindClusters using the first 20 principal components determined by the permutation-test-based jackstraw method ^[67]^, and a resolution parameter of 0.2. The clusters less than 10 cells were dislodged and doublets were removed using DoubletFinder package ^[68]^. t-Distributed Stochastic Neighbor Embedding (t-SNE) were used to visualize data in reduced dimensions ^[69]^. Marker genes across cell clusters were identified with the FindMarkers function of Seurat under the following criteria: 1, avg_log2FC > 0.5; 2, p-value < 0.05; and 3, min. pct > 0.7. The cell-type status of each group was determined on the basis of automated cluster annotation by SingleR package combined with top 20 marker genes of the highest avg_log2FC.

**Pseudotime analysis**

We applied the Single-Cell Trajectories analysis utilizing Monocle2 for SCs pseudotime analysis ^[70]^. we calculated the pseudotime for each cell and applied differentialGeneTest function to find marker genes that altered along pseudotime. Then standard reduceDimension and orderCells functions were performed to generate the trajectories, and the marker gene expression for each cluster was visualized.

**CellChat analysis**

CellChat ^[71]^ was used to evaluate the potential for cell-cell interactions in 2 datasets respectively, including regenerated pulp after 3- and 6- months implantation. CellChat was performed using default parameters.

**RNA-seq and bioinformatics**

Total RNA was extracted from rat primary TGNs using TRIzol (Invitrogen) according to the manufacturer's instructions. Subsequently, the concentration and quality of the total RNA were evaluated using a Nano Drop and an Agilent 2100 bioanalyzer (Thermo Fisher Scientific). Then, the mRNA was enriched by removing ribosomal RNA, digested and reverse‐transcribed into second‐strand cDNA. The cDNA library construction and sequencing were performed by Tsingke Biotechnology (China). The resulting high‐quality raw sequencing reads were mapped to the rat genome (mRatBN7.2) using the Hisat2 alignment tool (https://ccb.jhu.edu/software/hisat2/index.shtml). The gene expression level was normalized by fragments per kilobase of transcript per million mapped reads (FPKM). Significantly differentially expressed RNAs with the absolute value of fold change greater than 1.5 and P value less than 0.05 were annotated to Gene Ontology (GO) (https://www.geneontology.org/) pathways.

**4D label-free quantitative proteomic analysis**

Proteomic profiling was performed on TGN-EVs and ST-EVs using liquid chromatography tandem mass spectrometry (LC-MS) analysis carrier out by Lc-Bio Technologies (Hangzhou, China). EVs were lysed with lysis buffer (8M urea, 100mM TEAB, pH 8.5) and alkylated with sufficient iodoacetamide for 1 hour at room temperature in the dark. Trypsin was added to digest overnight at 37 ℃. The tryptic peptides were desalted using a C18 column (Waters BEH C18, 4.6×250 mm, 5 μm). The eluates were collected and dried by vacuum centrifugation.

**UHPLC-MS/MS analysis**

NanoElute UHPLC system (Bruker, Germany) coupled with a tims TOF pro2 mass spectrometer (Bruker, Germany) were used. The lyophilized powder was dissolved in 10 μL of mobile phase A (100% water, 0.1% formic acid) and centrifugated (4 ℃, 14,000g for 20min). The supernatant was transferred into the Liquid chromatography-mass spectrometry system for detection. The peptides were loaded onto a home-made analytical column (15 cm×100 μm, 1.9 μm) at a flow rate of 300 nL/min. Peptides were eluted from the column by linear gradient from 2% solvent B (100% acetonitrile, 0.1% formic acid) to 22% solvent B for 45 min; 35% solvent B for 5 min; 80% solvent B for 7 min; and hold for 5 min. The full scan range was from 100 to 1700 m/z and the Ramp time was 100 ms. The PASEF was set with 10 MS/MS scan with a total cycle time of 1.17 seconds.

The protein identification and quantitation were analyzed by Proteome Discoverer (Thermo, HFX and 480). The search parameters were set as follow: mass tolerance of 10 ppm for MS and 0.02 Da for production, carbamidomethyl on Cys was specified as fixed modification, methionine oxidation as dynamic modifications and acetylation as N-Terminal modification with a maximum of 2 missed cleavage sites were allowed. The false discovery rate for peptide-spectrum matches (PSMs) was set to no more than 0.01.

**Statistical analysis**

Statistical analysis was performed with Graphpad Prism 8.0 (GraphPad Software Inc., USA). The normality and equal variances of data were assessed before comparison. For normally distributed data with equal variances, non-parametric and unpaired wise comparisons were made with Student’s t test and multiple samples were compared using one-way ANOVA with Tukey’s correction for multiple comparison. The normally distributed data with unequal variances were analyzed using Welch’s t test, Welch ANOVA and the non-normal distributed data using Kruskal-Wallis test. For all analysis, Data were presented as mean±SD. P＜0.05 was considered as the level of significance.

**REFERENCES**

[61]	Shang F, Ming L, Zhou Z, et al. The effect of licochalcone a on cell-aggregates ecm secretion and osteogenic differentiation during bone formation in metaphyseal defects in ovariectomized rats. Biomaterials, 2014, 35: 2789-2797.

[62]	Malin SA, Davis BM, Molliver DC. Production of dissociated sensory neuron cultures and considerations for their use in studying neuronal function and plasticity. Nat Protoc, 2007, 2: 152-160.

[63]	Karai L, Brown DC, Mannes AJ, et al. Deletion of vanilloid receptor 1-expressing primary afferent neurons for pain control. J Clin Invest, 2004, 113: 1344-1352.

[64]	Weiss T, Taschner-Mandl S, Janker L, et al. Schwann cell plasticity regulates neuroblastic tumor cell differentiation via epidermal growth factor-like protein 8. Nat Commun, 2021, 12: 1624.

[65]	Hao Y, Hao S, Andersen-Nissen E, et al. Integrated analysis of multimodal single-cell data. Cell, 2021, 184: 3573-3587 e3529.

[66]	Korsunsky I, Millard N, Fan J, et al. Fast, sensitive and accurate integration of single-cell data with harmony. Nat Methods, 2019, 16: 1289-1296.

[67]	Chung NC, Storey JD. Statistical significance of variables driving systematic variation in high-dimensional data. Bioinformatics, 2015, 31: 545-554.

[68]	McGinnis CS, Murrow LM, Gartner ZJ. Doubletfinder: Doublet detection in single-cell rna sequencing data using artificial nearest neighbors. Cell Syst, 2019, 8: 329-337 e324.

[69]	Zhou B, Jin W. Visualization of single cell rna-seq data using t-sne in r. Methods Mol Biol, 2020, 2117: 159-167.

[70]	Trapnell C, Cacchiarelli D, Grimsby J, et al. The dynamics and regulators of cell fate decisions are revealed by pseudotemporal ordering of single cells. Nat Biotechnol, 2014, 32: 381-386.

[71]	Jin S, Guerrero-Juarez CF, Zhang L, et al. Inference and analysis of cell-cell communication using cellchat. Nat Commun, 2021, 12: 1088.

**Figure S1 to S10**

**Figure. S1. SHED implantation regenerates dental pulp with SCs enwrapped nerves.** (A) Immunostaining of MBP (myelin basic protein) and S100b of SCs in normal and regenerated dental pulps at 6 months after SHED implantation. White arrowheads show dental pulp nerves. Scale bar, 50 μm. Error bars denote SD from three minipig dental pulps per condition. Statistics by unpaired t test. ns, no significance. MFI, mean fluorescence intensity. **(B)** Immunostaining for co-localization of axons (βIII Tub) with CGRP (left panel), TRPV1 (center panel) and TRPM8 (right panel) in normal and regenerated dental pulps at 6 months after SHED implantation. White arrowheads show dental pulp nerves. Scale bar, 50 μm. **(C)** Heatmap of representative featured top 10 gene expression in different cell clusters.

**Figure. S2. Implanted SHED differentiated into SCs, MSCs, DPSCs and endothelium. (A)** Representative featured gene expression in SCs, MSCs, DPCs and endothelium. **(B)** Immunostaining for co-localization of GFP^+^ SHED (green) with S100b (left panel), CD31 (center panel) and DSPP (right panel) in normal and regenerated dental pulp at 6 months after GFP-SHED implantation. White arrowheads showed SCs, endothelium and odontoblast layer in dental pulp. Scale bar, 50 μm.

**Figure. S3. Implanted SHED integrate with host cellular component to regenerate dental pulp. (A)** Spline plots showing the expression of marker genes across pseudotime in MSCs, SCs and m-SCs. **(B and C)** CellChat analysis of intercellular communication network of secreted and received signals in regenerated dental pulp at 3 and 6 months after SHED implantation. **(D and E)** The role of various cells in the regenerated dental pulp as signal transmitters and receivers in multiple signaling pathways.

**Figure. S4. SHED**-**EVs trigger host axonal growth and dental nerve regeneration. (A and B)** Immunostaining (A) and quantification (B) for S100b positive SCs and MBP positive SCs at 6 months after DMSO-SHED or GW4869-SHED implantation, as showed by white arrowheads. Scale bar, 50 μm. Error bars denote SD from three regenerated minipig dental pulps per condition. Statistics by unpaired *t* test. **(C)** HE staining for normal (left panel) and regenerated dental pulps (right panel) at 6 weeks after SHED implantation in rats. Black dotted lines show odontoblast layer and black arrowheads show blood vessels. Scale bar, 100 μm. **(D and E)** Normal and regenerated dental pulps in rats stained for βIII Tub, GFAP, h-mit and DAPI (D). The fluorescence intensity profiles (E) for GFAP (black), βIII Tub (green) and h-mit (red) from the solid yellow line. White arrowheads in left two panel show SCs in normal and regenerated rat dental pulps. The white box shows the magnified regions. Scale bar, 20 μm in the left and 8 μm in the right panel. **(F and G)** Ex vivo fluorescent images (F) and quantification (G) of TGs injected with DiR-labeled SHED-EVs (yellow arrowheads) or PBS (white arrowheads) into root canal at 3 and 5 days after injection. Error bars denote SD from four rats per condition. Statistics by unpaired *t* test.

**Figure. S5. SHED-EVs trigger dental nerve regeneration in rats. (A)** Immunostaining revealed the internalization of PKH26-labeled SHED-EVs (red) by the TGN. SHED-EVs PKH26 were incubated with TGN at DIV3 for 24 h and 48 h, followed by immunostaining for βIII Tub (green). White box shows the enlarged region of the image. Scale bar, 50 μm in left panel and 10 μm in right panel. **(B)** Volcano plot analysis identifying the upregulated and downregulated genes in TGN treated with SHED-EVs. **(C)** The GO analysis for Biological Process, Molecular Function, and Cellular Component of both up- and down-regulated genes in TGN. (D to F) The effect of GW4869-SHED co-culture on axonal growth of TGN. Schematic drawings of transwell co-culture of GW4869-SHED or DMSO-SHED with DIV2 primary TGN for 24 h (D). Immunostaining (E) and quantification (F) of axonal length of TGN. Scale bar, 25 μm. Error bars denote SD from thirty TGN per condition. Statistics by unpaired t test. (G to I) The effect of shRAB27A-SHED co-culture on axonal growth of TGN. Schematic drawings of transwell co-culture of shRAB27A-SHED or NC-SHED with DIV2 primary TGN for 24 h (G). Immunostaining (H) and quantification (I) of TGN axonal length. Scale bar, 25 μm. Error bars denote SD from thirty TGN per condition. Statistics by Welch’s t test.

**Figure. S6. Reciprocal EVs communication between donor SHED and host TG in dental nerve regeneration.** **(A and B)** Immunostaining (A) and quantification (B) of regenerated axons and SC differentiation, as identified by S100 and MBP in direct co-culture of SHED and TGN for 2, 4, 6 and 8 weeks. Scale bar, 100 μm. Error bars denote SD from six images per condition over three independent experiments. Statistics by one-way ANOVA with Tukey’s multiple comparison analysis. **(C and D)** Immunostaining (C) and quantification (D) of regenerated axons and SC differentiation, as identified by S100 and MBP in direct co-culture of SHED and TGN after subcutaneously implanted into immunocompromised mice at 2, 4, 6 and 8 weeks. SHED were co-cultured with TGN for 1 week before implantation. Scale bar, 100 μm. Error bars denote SD from six immunocompromised mice per condition. Statistics by one-way ANOVA with Tukey’s multiple comparison analysis. **(E and F)** Immunostaining (E) and quantification (F) of regenerated axons and SC differentiation as identified by S100b and MBP in direct co-culture of TGN and SHED for 6 weeks. shRab27a was transfected into TGN, whereas SHED were transfected with shRAB27A to inhibit EV secretion. Scale bar, 100 μm. Error bars denote SD from six images per condition over three independent experiments. Statistics by one-way ANOVA with Kruskal-Wallis test for βIII Tub intensity, Brown-Forsythe and Welch ANOVA for S100b/βIII Tub and one-way ANOVA with Tukey’s multiple comparison analysis for MBP/βIII Tub.

**Figure. S7. EVs from TGN after internalization of SHED-EVs promote the differentiation of SHED into SCs** **in vitro.** **(A)** Western blot analysis for the expression of TGN-EVs positive markers CD9, CD63, TSG101 and Alix and positive marker Calnexin. **(B)** Schematic drawings of PKH67-labeled TGN-EVs (green) and PKH26-labeled SHED-EVs internalization in SHED and TGN. **(C)** Immunostaining of PKH67-labeled TGN-EVs (green) internalized by SHED and TGN, which was identified by CD90 (top panel, red) and βIII Tub (bottom panel, red). Scale bar, 10 μm. **(D)** Immunostaining of PKH26-labeled SHED-EVs (red) internalized by SHED and TGN, which was identified by CD90 (top panel, green) and βIII Tub (bottom panel, green) (F). Scale bar, 10 μm. **(E)** Quantification of engulfed TGN-EVs (green fluorescence intensity) in SHED and TGN. The fluorescence intensity was normalized to TGN. Error bars denote SD from thirty SHED and TGN. Statistics by Welch’s *t* test. **(F)**. Quantification of engulfed SHED-EVs (red fluorescence intensity) in SHED and TGN. The fluorescence intensity was normalized to SHED. Error bars denote SD from thirty SHED and TGN. Statistics by unpaired *t* test.

**Figure. S8. ST-EVs enriched with TUFM and GFM2 promote the differentiation of SHED into SCs. (A and B)** The characterization of ST-EVs, TEM (A) and NTA analysis (B) of ST-EVs. Scale bar, 100 nm. **(C)** Volcano plot analysis identifying both the upregulated and downregulated proteins in ST-EVs compared with TGN-EVs. **(D and E)** Immunostaining (D) and quantification (E) of SC differentiation (MBP) in direct co-culture of SHED and TGN. SHED were pre-treated with SHED-EVs, TGN-EVs and ST-EVs at same concentration with 30μg/ml and co-cultured with TGN for 6 weeks. Scale bar, 100 μm. Error bars denote SD from six images per condition over three independent experiments. Statistics by one-way ANOVA with Tukey’s multiple comparison analysis. **(F and G)** Immunostaining (F) and quantification (G) of SC differentiation (MBP) in co-cultured SHED and TGN after subcutaneously implanted into immunocompromised mice for 6 weeks. SHED were pre-treated with SHED-EVs, TGN-EVs and ST-EVs and co-cultured with TGN for 1 week before implantation. Scale bar, 100 μm. Error bars denote SD from six immunocompromised mice per condition. Statistics by one-way ANOVA with Tukey’s multiple comparison analysis. **(H and I)** Immunostaining (H) and quantification (I) of the co-localization between TOMM20 and CD63 in soma and axon of TGN stimulated by SHED-EVs after 12 h and 24 h. Scale bar, 10 μm. Error bars denote SD from thirty TGN per condition. Statistics by one-way analysis of variance (ANOVA) with Kruskal-Wallis test for TOMM20 in soma and one-way ANOVA with Tukey’s multiple comparison analysis for TOMM20 in axon. **(J and K)** Immunostaining (J) and quantification (K) of the co-localization between TOMM20 and CD63 in rat TG after implantation of SHED into maxillary first molars. The dental pulp was removed in control group and the regeneration group underwent SHED implantation. Scale bar, 200 μm in soma and 100 μm in axon. Error bars denote SD from four rat TGs per condition. Statistics by unpaired t test.

**Figure. S9. The homology of TUFM and GFM2 in human and rat. (A)** The predicted protein structure of human TUFM and rat Tufm (top panel), human GFM2 and rat Gfm2 (bottom panel) by AlphaFoldDB modeling approach. Red arrows show N-terminal extension structure. **(B)** The expression of TUFM and GFM2 in ST-EVs, ST-EVs^shTufm^ and ST-EVs^shGfm2^ by western blot.

**Figure. S10. ST-EVs transfer TUFM to promote SC differentiation of SHED. (A and B)** OCR traces during seahorse extracellular flux experiment (A) and quantification (B) of basal respiration, ATP production and maximal respiration. NC, negative control SHED. Error bars denote SD from four independent samples per condition. Statistics by one-way ANOVA with Tukey’s multiple comparison analysis. **(C to E)** SC differentiation of NC-SHED or shTUFM-SHED were treated with PBS and ST-EVs (30μg/ml). The morphology was detected by Phalloidine staining (C) and analyzed with aspect ratio (D) and the SCs-related makers were measured by western blot (E). Scale bar, 100 μm. Error bars denote SD from thirty SHED per condition. Statistics by one-way ANOVA with Tukey’s multiple comparison analysis. **(F and H)** Immunostaining (F) and quantification (H) of SC differentiation, as identified by S100 and MBP in direct co-culture of SHED and TGN. NC-SHED or shTUFM-SHED pretreated with PBS and ST-EVs were co-cultured with TGN for 6 weeks. Scale bar, 100 μm. Error bars denote SD from six images per condition over three independent experiments. Statistics by one-way ANOVA with Tukey’s multiple comparison analysis for S100b/βIII Tub and Brown-Forsythe and Welch ANOVA for MBP/βIII Tub. **(G and I)** Immunostaining (G) and quantification (I) of SC differentiation, as identified by S100 and MBP in co-cultured SHED and TGN subcutaneously implanted into immunocompromised mice with empty root canal for 6 weeks. Negative control SHED (NC) or shTUFM-SHED pretreated with PBS and ST-EVs (30μg/ml) were co-cultured with TGN for 1 week before implantation. Scale bar, 100 μm. Error bars denote SD from six immunocompromised mice per condition. Statistics by one-way ANOVA with Tukey’s multiple comparison analysis. **(J to L)** The effect of TUFM overexpression on SC differentiation of SHED. The SC-related markers expression was detected by western blot (J). Immunofluorescence staining Immunostaining (K) and quantification (L) of SC differentiation in direct co-culture of SHED and TGN. NC SHED or OE TUFM-SHED were co-cultured with TGN for 6 weeks. Scale bar, 100 μm. Error bars denote SD from six images per condition over three independent experiments. Statistics by unpaired t test.

**Tables S1 and S2**

**Table S1. The similarity of protein sequence of TUFM and GFM2 in human and rat**

sp|P49411|EFTU_HUMAN MTTMAAATLLRATPHFSGLAAGRTFLLQGLLRLLKAPALPLLCRGLAVEAKKTYVRDKPH 60

VNVGTIGHVDHGKTTLTAAITKILAEGGGAKFKKYEEIDNAPEERARGITINAAHVEYST 120

AARHYAHTDCPGHADYVKNMITGTAPLDGCILVVAANDGPMPQTREHLLLARQIGVEHVV 180

VYVNKADAVQDSEMVELVELEIRELLTEFGYKGEETPVIVGSALCALEGRDPELGLKSVQ 240

KLLDAVDTYIPVPARDLEKPFLLPVEAVYSVPGRGTVVTGTLERGILKKGDECELLGHSK 300

NIRTVVTGIEMFHKSLERAEAGDNLGALVRGLKREDLRRGLVMVKPGSIKPHQKVEAQVY 360

ILSKEEGGRHKPFVSHFMPVMFSLTWDMACRIILPPEKELAMPGEDLKFNLILRQPMILE 420 KGQRFTLRDGNRTIGTGLVTNTLAMTEEEKNIKWG	455

sp|P85834|EFTU_RAT

MAAATLLRATPRFSGLCASPTPFLQGRLRPLKAPASPFLCRGLAVEAKKTYVRDKPH 57 VNVGTIGHVDHGKTTLTAAITKILAEGGGAKFKKYEEIDNAPEERARGITINAAHVEYST 117

AARHYAHTDCPGHADYVKNMITGTAPLDGCILVVAANDGPMPQTREHLLLAKQIGVEHVV 177

VYVNKADAVQDSEMVELVELEIRELLTEFGYKGEETPVIVGSALCALEQRDPELGVKSVQ 237

KLLDAVDTYIPVPTRDLEKPFLLPVESVYSIPGRGTVVTGTLERGILKKGDECELLGHNK 297

NIRTVVTGIEMFHKSLERAEAGDNLGALVRGLKREDLRRGLVMVKPGSIQPHQKVEAQVY 357

ILSKEEGGRHKPFVSHFMPVMFSLTWDMACRVILPPGKELAMPGEDLKLSLILRQPMILE 417

KGQRFTLRDGNKTIGTGLVTDVPAMTEEDKNIKWS	452

sp|Q969S9|RRF2M_HUMAN MLTNLRIFAMSHQTIPSVYINNICCYKIRASLKRLKPHVPLGRNCSSLPGLIGNDIKSLH 60

SIINPPIAKIRNIGIMAHIDAGKTTTTERILYYSGYTRSLGDVDDGDTVTDFMAQERERG 120 ITIQSAAVTFDWKGYRVNLIDTPGHVDFTLEVERCLRVLDGAVAVFDASAGVEAQTLTVW 180

RQADKHNIPRICFLNKMDKTGASFKYAVESIREKLKAKPLLLQLPIGEAKTFKGVVDVVM 240

KEKLLWNCNSNDGKDFERKPLLEMNDPELLKETTEARNALIEQVADLDDEFADLVLEEFS 300

ENFDLLPAEKLQTAIHRVTLAQTAVPVLCGSALKNKGIQPLLDAVTMYLPSPEERNYEFL 360

QWYKDDLCALAFKVLHDKQRGPLVFMRIYSGTIKPQLAIHNINGNCTERISRLLLPFADQ 420

HVEIPSLTAGNIALTVGLKHTATGDTIVSSKSSALAAARRAEREGEKKHRQNNEAERLLL 480

AGVEIPEPVFFCTIEPPSLSKQPDLEHALKCLQREDPSLKVRLDPDSGQTVLCGMGELHI 540

EIIHDRIKREYGLETYLGPLQVAYRETILNSVRATDTLDRTLGDKRHLVTVEVEARPIET 600

SSVMPVIEFEYAESINEGLLKVSQEAIENGIHSACLQGPLLGSPIQDVAITLHSLTIHPG 660 TSTTMISACVSRCVQKALKKADKQVLEPLMNLEVTVARDYLSPVLADLAQRRGNIQEIQT 720

RQDNKVVIGFVPLAEIMGYSTVLRTLTSGSATFALELSTYQAMNPQDQNTLLNRRSGLT- 779

sp|Q5BJP6|RRF2M_RAT MFTNWRIFAVNRQKTFSVHIHTTCYCKIKANLKRSKTQVPLTRSYSSPPGIVGNEVKSLH 60

SIINPPVAKIRNIGIMAHIDAGKTTTTERILYYSGYTRSLGDVDDGDTVTDFMAQERERG 120

ITIQSAAVTLDWKGYRVNLIDTPGHVDFTLEVERCLRVLDGAVAVFDASAGVEAQTLTVW 180 RQADKHKIPRICFLNKMDKTGASFNYAVESIREKLKAKPLILQLPIGEARTFQGVVDVVN 240 REKLIWNSDSDDGKDFERKPLSEASDPTLLKETVEARNSLIEQVADLDDEFADLVLGEFS 300 EDFDLVPAEKLQAAIHRVTLAQAAVPVLCGSALKNKGVQPLLDAVTTYLPSPEEREHGFL 360 QWYKGDLCALAFKVLHDKQRGPLVFLRIYSGTLTPQSAVHNVNRNCTERMSRLLLPFADQ 420 HVEIPSLTAGNIALTVGLKQTATGDTIVSSKSSALAAARRAGK-GERKPGRISEAESVLL 479 AGVEIPEPVFFCTIEPPSAAKQPDLDHALEHLQREDPSLKVKLDPDSGQTVLCGMGELHI 539 EIIHDRIKREYGLETYLGPLQVAYRETILNSVRATDTLDRVLGDKRHFARAELEVRPAEE 599

PCGVATIEYADSVGEDMLQAPREDIENAIHSACLQGPLLGSPIQDVAVTLHSLMIHPG 657 TSTTMVTACISRCVQKALKKADKQVLEPLMSLEVTVSREYLSPVLADLAQRRGNIQEIQT 717 RQDNKVVLGFVPLAEIMGYSTVLRTLTSGSATFALELSTYQAMSPQDQRTLLSQRSGLARVL 779

**Table S2. Reagents and resources**

| **REAGENT or RESOURCE** | **SOURCE** | **IDENTIFIER** |
| --- | --- | --- |
| **Antibodies** |  |  |
| Rabbit Anti-S100b Antibody | Abcam | ab52642 |
| Rabbit Anti-TUFM Antibody | Abcam | ab173300 |
| Rabbit Anti-TSG101 Antibody | Abcam | ab125011 |
| Rabbit Anti-TOMM20 Antibody | Abcam | ab186735 |
| Mouse Anti-GFP Antibody | Abcam | ab1218 |
| Mouse Anti-betaIII Tubulin Antibody | Abcam | ab78078 |
| Rabbit Anti-betaIII Tubulin Antibody | Abcam | ab18207 |
| Rabbit Anti-mCherry Antibody | Abcam | ab167453 |
| Mouse Anti-CD90 Antibody | Abcam | ab181469 |
| Mouse Anti-human mitochondria Antibody | Abcam | ab215892 |
| Rabbit Anti-TRPV1 Antibody | Abcam | ab305299 |
| Rabbit Anti-CD31 Antibody | Abcam | ab28364 |
| Rabbit Anti-DSPP Antibody | Abcam | ab216892 |
| Rabbit Anti-p75NTR Antibody | Cell Signaling Technology | 8238 |
| Rabbit Anti-GFAP Antibody | Cell Signaling Technology | 80788 |
| Rabbit Anti-MBP Antibody | Cell Signaling Technology | 78896 |
| Rabbit Anti-CGRP Antibody | Cell Signaling Technology | 14959 |
| Mouse Anti-CD63 Antibody | Proteintech | 67605-1-Ig |
| Rabbit Anti-TRPM8 Antibody | Proteintech | 12813-1-AP |
| Rabbit Anti-beta actin Antibody | Proteintech | 81115-1-RR |
| Rabbit Anti-GFM2 Antibody | Huabio | ER1909-51; |
| Rabbit Anti-CD9 Antibody | Huabio | ET1601-9 |
| Chicken Anti-GFAP Antibody | Merckmillipore | AB5541 |
| Alexa Fluor 647 goat anti-Chicken  IgG (H+L) Antibody | Abcam | ab150171 |
| HRP-linked goat anti-rabbit IgG,  Antibody | Cell Signaling Technology | 7076 |
| HRP-linked goat anti-rabbit IgG,  Antibody | Cell Signaling Technology | 7074 |
| FITC AffiniPure Goat Anti-Rabbit IgG (H+L) | Yeasen | 33107ES60 |
| Alexa Flour ® 488 Goat Anti-Mouse IgG | Yeasen | 33206ES60 |
| Cy™3 AffiniPure Goat Anti-Rabbit IgG (H+L) | Yeasen | 33108ES60 |
| Alexa Flour ® 594Goat Anti-Mouse IgG | Yeasen | 33212ES60 |
| **Chemicals and reagent** |  |  |
| a-MEM | Gibco | 2110309 |
| DMEM | Gibco | 31600083 |
| FBS | Gibco | 10099141C |
| Vitamin C | Sigma | A92902 |
| Neuralbasal medium | Gibco | 21103049 |
| Glutamax | Gibco | 35050079 |
| B27 | Gibco | A1486701 |
| Collagenase Type I | Gibco | 17018029 |
| Collagenase Type IV | Gibco | 17104019 |
| Trypsin | Gibco | 25200056 |
| Puromycin | MedChemExpress | HY-B1743A |
| GW4869 | MedChemExpress | HY-19363 |
| Dynasore | MedChemExpress | HY-15304 |
| DiR | Invitrogen | D12731 |
| Phalloidin | Yeasen | 40734ES75 |
| DAPI | Yeasen | 40728ES03 |
| Hoechst 33342 | Yeasen | 40731ES10 |
| Penicillin/Streptomycin | Gibco | 15070063 |
| Triton X-100 | MP Biomedicals | 194854 |
| Poly-L-Lysine | Solarbio | P8140 |
| Paraformaldehyde | Solarbio | P1110 |
| EDTA | MP Biomedicals | 0219482290 |
| **Kits** |  |  |
| Seahorse XF Cell Mito Stress Test | Agilent | 103708-100 |
| PKH26 | Sigma | MINI26-1KT |
| PKH67 | Sigma | MINI67-1KT |
